# A study of animal action segmentation algorithms across supervised, unsupervised, and semi-supervised learning paradigms

**Published:** 2024-07-23

**Authors:** Ari Blau, Evan S Schaffer, Neeli Mishra, Nathaniel J Miska, The International Brain Laboratory, Liam Paninski, Matthew R Whiteway

**Affiliations:** Department of Statistics Columbia University; Icahn School of Medicine Mount Sinai; Zuckerman Institute Columbia University; Sainsbury Wellcome Centre University College London; International Brain Laboratory; Department of Statistics Zuckerman Institute Columbia University; Zuckerman Institute Columbia University

## Abstract

Action segmentation of behavioral videos is the process of labeling each frame as belonging to one or more discrete classes, and is a crucial component of many studies that investigate animal behavior. A wide range of algorithms exist to automatically parse discrete animal behavior, encompassing supervised, unsupervised, and semi-supervised learning paradigms. These algorithms — which include tree-based models, deep neural networks, and graphical models — differ widely in their structure and assumptions on the data. Using four datasets spanning multiple species — fly, mouse, and human — we systematically study how the outputs of these various algorithms align with manually annotated behaviors of interest. Along the way, we introduce a semi-supervised action segmentation model that bridges the gap between supervised deep neural networks and unsupervised graphical models. We find that fully supervised temporal convolutional networks with the addition of temporal information in the observations perform the best on our supervised metrics across all datasets.

## 1 Introduction

**Figure 1:**
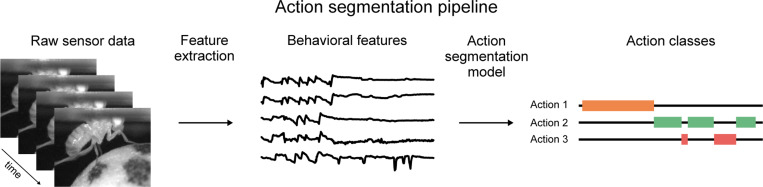
Overview of the action segmentation pipeline. Raw sensor data (e.g. video) is collected, then features are extracted (e.g. pose estimates), then an action segmentation model is trained to map those features to a discrete behavioral class for each frame.

Action segmentation of video data is a process that classifies discrete animal behaviors given a set of spatiotemporal video features. It is an indispensable tool for quantifying natural animal behavior across a range of experimental paradigms, from spontaneous behaviors in open arenas to complex social interactions (Anderson et al. 2014; Pereira et al. 2020; Ziegler et al. 2021). This procedure begins with the collection of raw behavioral data during an experiment, typically with video cameras or motion-capture sensors. The next step is to reduce the dimensionality of the video data using pose estimation (Mathis et al. 2018; Pereira et al. 2019; Biderman et al. 2024), autoencoders (Batty et al. 2019; Whiteway et al. 2021), or other techniques (Sun et al. 2020; Bohnslav et al. 2021). Finally, an action segmentation model finds discrete behaviors from the low-dimensional representation (Fig. 1).

**Figure 2:**
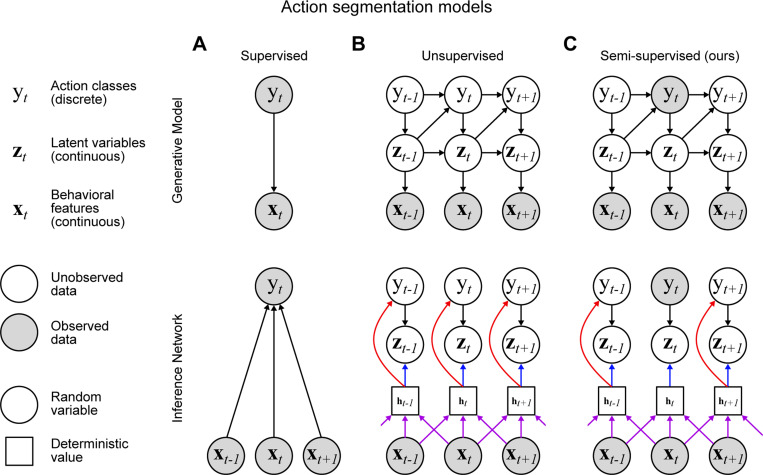
Overview of action segmentation models. **A:** *Top*: Graphical model for supervised classification. Both discrete states $y_{t}$ and poses $x_{t}$ are observed. *Bottom*: Inference network for the supervised model. We use a window of observed behavioral features for state prediction. **B:** *Top*: Graphical model for an unsupervised recurrent switching dynamical system. The set of discrete states {$y_{t}$ } and continuous latents {$z_{t}$} are unobserved. *Bottom*: The inference network uses a window of observed behavioral features to create a deterministic hidden representation $h_{t}$ (purple arrows); this is then used to predict the continuous latents $z_{t}$ (blue arrows) and discrete latents $y_{t}$ (red arrows). Note that the purple and red arrows together define a classifier for the discrete state at each time step. **C:** Graphical model and inference network for a semi-supervised recurrent switching dynamical system. A subset of the discrete states are observed. During inference, the observed discrete state is used for the inference of $z_{t}$ when possible.

Supervised action segmentation (Fig. 2A) requires the experimenter to annotate a subset of frames that contain behaviors of interest, such as grooming or sniffing. Then a classifier is trained to match each frame (or its low-dimensional representation) with the corresponding label (Jhuang et al. 2010; Kabra et al. 2013; Kramida et al. 2016; Le et al. 2019; Murari et al. 2019; Nguyen et al. 2019; Dam et al. 2020; Sturman et al. 2020; Bohnslav et al. 2021; Segalin et al. 2021; Gabriel et al. 2022; Goodwin et al. 2024). As the scale of behavioral data continues to grow (Gomez-Marin et al. 2014; Ziegler et al. 2021), it becomes infeasible to densely label behaviors in every video. Therefore, it is crucial to develop action segmentation models that perform well with sparsely labeled data. Furthermore, we would like to develop techniques that also take advantage of the vast amounts of unlabeled data.

Unsupervised action segmentation models (Fig. 2B) are a complementary approach that do not require any hand labels (Berman et al. 2014; Wiltschko et al. 2015; Johnson et al. 2016; Hsu et al. 2021; Luxem et al. 2022; Weinreb et al. 2024) and perform clustering on the low-dimensional behavioral representation (Datta et al. 2019). These unsupervised models are more scalable than their supervised counterparts since they do not require annotations for training. Another benefit of this approach is its ability to discover new behaviors that are not pre-defined by the experimenter (Datta et al. 2019; Pereira et al. 2020). However, there may be certain behaviors of particular interest to downstream users, and unsupervised models cannot guarantee that they cluster these behaviors accurately.

The recent proliferation of approaches to animal action segmentation raises obvious practical questions: how do these approaches compare, and what are their trade-offs? To aid in this endeavor, we introduce an action segmentation model that bridges the gap between supervised deep neural networks and unsupervised graphical models through the use of semi-supervised state space models (Fig. 2C). Graphical models, particularly switching linear dynamical systems, are an increasingly ubiquitous approach to unsupervised animal action segmentation (Wiltschko et al. 2015; Johnson et al. 2016; Buchanan et al. 2017; Costacurta et al. 2022; Weinreb et al. 2024). These models posit that each discrete action is defined by a set of linear dynamics, and infer which set of dynamics best describes the observations at each time point (Datta et al. 2019). We utilize such a model and bias it towards interpretable solutions by providing a small set of hand labels during training. Our inference procedure leads to a deep neural network classifier that allows us to make direct comparisons with fully supervised classifiers. We refer to the resulting model and inference technique as a semi-supervised switching linear dynamical system (S^3^LDS).

Another important consideration for the animal action segmentation problem is the behavioral representation used as input to a given model. Much previous work with supervised models uses a host of quantities derived from pose estimates, like distances and angles between different keypoints (Sturman et al. 2020; Segalin et al. 2021; Goodwin et al. 2024). Much of the unsupervised literature simply uses the pose estimates themselves (perhaps converting to egocentric coordinates first) and allow the model to perform the necessary transformations (Luxem et al. 2022; Weinreb et al. 2024) (though see Berman et al. 2014; Hsu et al. 2021 for approaches that involve feature engineering). We would also like to know how simple choices about the behavioral representation – such as whether or not temporal information contained in velocity or acceleration measurements is included – impacts the performance of these models.

To address these questions we use an array of models, including S^3^LDS, to evaluate four behavioral datasets: a head-fixed fly spontaneously behaving on an air-supported ball (Schaffer et al. 2023), a mouse freely moving around an open arena (Sturman et al. 2020), a head-fixed mouse performing a perceptual decision-making task from the International Brain Laboratory (IBL et al. 2023), and a human gait dataset (HuGaDB) (Chereshnev et al. 2017). For the first three datasets, we experiment with both position features (static pose information) and position-velocity features (including the velocity of each static keypoint). The HuGaDB data is comprised of three dimensional accelerometer and gyroscope data collected from inertial measurement units (IMUs).

It is challenging to comprehensively compare the performance of such a wide range of models, and we therefore limit ourselves to evaluating how much the outputs of the models align with human-annotated behaviors. This supervised metric will of course miss many of the nuances provided by these models, especially those which are fully unsupervised. We find that the S^3^LDS offers improvements over supervised models when using the position features as input, but these advantages disappear once we include the velocity features. In the latter case, a fully supervised temporal convolutional network (TCN) performs best across all datasets (Sun et al. 2021; Schaffer et al. 2023; Everett et al. 2024). We also compare the S^3^LDS and fully supervised models to Keypoint-MoSeq (Weinreb et al. 2024), a closely related yet fully unsupervised action segmentation method, and find that the models trained with discrete behavior labels learn an improved latent representation, as measured by several supervised cluster quality metrics.

## 2 Results

We first provide a brief overview of the different types of supervised models that we compare, and establish which one we use as our baseline. We explore how performance is affected by the choice of behavioral features used by the various models. Next, we introduce our proposed semi-supervised framework, S^3^LDS, which we compare to our supervised model using supervised classification metrics. We also perform several ablation experiments demonstrating which aspects of the semi-supervised model are most important for performance. Finally, we compare the supervised and semi-supervised models to Keypoint-MoSeq (Weinreb et al. 2024), an unsupervised model closely related to the S^3^LDS.

### 2.1 Comparing supervised baselines

We experiment with three fully supervised models: a temporal convolution network (TCN) (Lea et al. 2016; Lea et al. 2017), random forests (Breiman 2001), and XGBoost (Chen et al. 2016). TCNs have been competitive on human action segmentation and recognition benchmarks (Kim et al. 2017; Farha et al. 2019; Filtjens et al. 2022). The two tree-based methods are commonly used as supervised classifiers for animal action segmentation (Segalin et al. 2021; Goodwin et al. 2024).

Another common question in the literature is how to transform the data before model fitting. Previous work has proposed various hand-engineered features (Berman et al. 2014; Hsu et al. 2021; Segalin et al. 2021; Goodwin et al. 2024) and learned features (Sun et al. 2020; Bohnslav et al. 2021). While a thorough characterization of this choice is beyond the scope of this study, we test two scenarios: modeling with position features (no temporal information) versus position-velocity features (contains temporal information).

We find the TCN achieves the best performance with both feature types (Fig. S1). We also find that temporal information in the features is useful for all methods. We select the TCN to use as our baseline supervised model, not only because of its superior performance, but also because of its compatibility to function as an inference network in the deep graphical models we introduce next.

### 2.2 Introducing a semi-supervised action segmentation model

Next we move to semi-supervised models, which utilize both labeled and unlabeled data. We focus on the switching linear dynamical system (SLDS) and develop a new amortized variational inference scheme. The SLDS is commonly used to model complex nonlinear behavior in dynamical systems (Ackerson et al. 1970; Chang et al. 1978; Hamilton 1990). Several studies have used the SLDS (and the related auto-regressive hidden Markov model, or ARHMM) to model animal behavior (Wiltschko et al. 2015; Johnson et al. 2016; Buchanan et al. 2017; Markowitz et al. 2018; Batty et al. 2019; Wiltschko et al. 2020; Whiteway et al. 2021; Costacurta et al. 2022; Markowitz et al. 2023; Weinreb et al. 2024). The motivation to construct our semi-supervised model from the SLDS is two-fold: (1) the SLDS is typically utilized as an unsupervised model, and hence allows us to make easy connections between these two learning paradigms; (2) the amortized variational inference scheme will result in a classifier that can be directly compared to the supervised TCN model above.

The standard setup for the generative model of an SLDS is as follows (Fig. 2B): let $y_{t}\in \{0,\ldots ,K-1\}$ be the discrete behavioral state at time t with Markovian dynamics, i.e. the state at time t is only dependent on the state at time t−1. The observed data $x_{t}$ are modeled using piecewise-linear dynamics in a latent space denoted by $z_{t}$:

(1) $$p_{\theta }\left(y_{t}|y_{t-1}\right)=Cat\left(y_{t}|\pi_{\theta }\left(y_{t-1}\right)\right)$$

(2) $$p_{\theta }\left(z_{t}|z_{t-1},y_{t}\right)=N\left(z_{t}|A_{y_{t}}z_{t-1}+b_{y_{t}},Q_{y_{t}}\right)$$

(3) $$p_{\theta }\left(x_{t}|z_{t},y_{t}\right)=N\left(x_{t}|C_{y_{t}}z_{t}+d_{y_{t}},S_{y_{t}}\right),$$

where t∈{1,2,…,T}. $A_{y_{t}}$ and $b_{y_{t}}$ define the latent linear dynamics associated with state $y_{t}$; $C_{y_{t}}$ and $d_{y_{t}}$ represent the linear mapping from the continuous latents to the observations; and $Q_{y_{t}}$ and $S_{y_{t}}$ represent noise covariance matrices of the latent and observed data. In other words, at each time point, the model selects one set of the K linear dynamics (which each define a discrete action class; Eq. 1) that operate in a continuous latent space, evolves the latents forward in time using those linear dynamics (Eq. 2), then projects those latents to the observations (the behavioral features; Eq. 3).

The recurrent SLDS (rSLDS) builds on the SLDS by conditioning $y_{t}$ on $z_{t-1}$ in addition to $y_{t-1}$ (Linderman et al. 2016) (Fig. 2B). This recurrence allows the model to more flexibly switch between discrete states based on the trajectory through the latent space, rather than just static switching probabilities. The new generative model therefore replaces Eq. 1 with

(4) $$p_{\theta }\left(y_{t}|y_{t-1},z_{t-1}\right)=\text{Cat}\left(y_{t}|\pi_{\theta }\left(y_{t-1},z_{t-1}\right)\right).$$

In both the SLDS and recurrent SLDS, it is assumed the discrete action classes are all unobserved. We propose to allow the model access to labels for the discrete action classes for a small number of time points (Fig. 2C, *top*), resulting in a semi-supervised SLDS (S^3^LDS). We develop an efficient and flexible amortized variational inference strategy that incorporates both labeled and unlabeled data. Importantly, the proposed inference strategy leads to the definition of an approximate posterior $q_{\phi }\left(y_{t}|{\text{x}}_{1:T}\right)$ which also serves as a classifier (Willetts et al. 2020), predicting the discrete action class $y_{t}$ from the observed data $x_{1:T}$ (Fig. 2C, *bottom*). We implement this approximate posterior using the TCN model of the previous section, allowing us to directly assess the benefits of training this classifier network with both labeled and unlabeled data. For more details, see the Methods section.

### 2.3 Semi-supervised and supervised model comparisons

We start by comparing supervised and semi-supervised models using a supervised classification metric, and report several ablation studies along the way that highlight the role of various components of the S^3^LDS. Due to our choice of the TCN for the S^3^LDS inference network, we also use a TCN with the exact same architecture as our supervised baseline.

**Figure 3:**
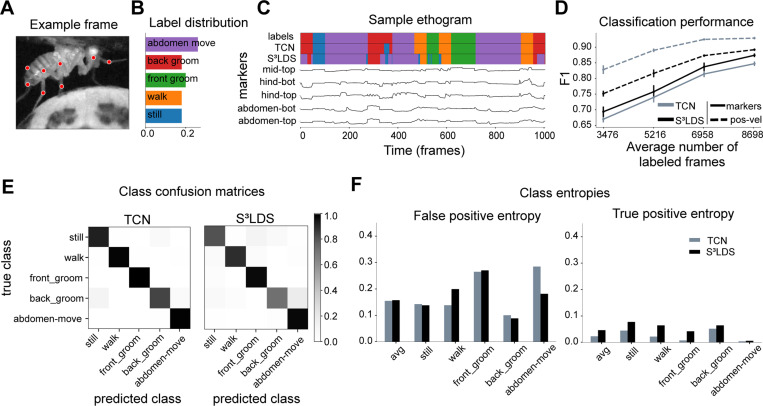
Supervised vs semi-supervised results for the head-fixed fly. **A:** Example frame of the fly, overlaid with pose markers. **B:** Proportion of each labeled behavior in the training dataset. **C:** Sample of ground truth labels, along with predictions from both the TCN and the S^3^LDS models. Below is a subset of the corresponding features used as inputs to the models. **D:** F1 scores for the TCN and S^3^LDS models. We show results for the position features (solid lines) as well as the position-velocity features (dashed lines). Adding velocity improves performance for both models. Error bars represent the standard deviation of the F1 scores over five subsamples of the training data. **E:** Confusion matrices for the TCN and S^3^LDS models. **F:** Average entropy of the false positives (left) and true positives (right) for both models. In both cases entropies are larger for the S^3^LDS model. These results are consistent with the other datasets as shown in Fig. S2. Panels E and F only show results from the models trained with position-velocity features.

We begin with the head-fixed fly dataset (Fig. 3A), which contains five annotated behaviors: still, walk, front groom, back groom, and abdomen move (Fig. 3B,C). We extract pose estimates from the videos using Lightning Pose and the Ensemble Kalman Smoother post-processor (Biderman et al. 2024), and use these position features as input to the TCN and S^3^LDS. We train models with an increasing number of labeled videos (five networks trained per condition, each using a random subset of labeled videos). The S^3^LDS uses all available unlabeled frames. We find that performance, as measured by F1 (the harmonic mean of accuracy and precision; higher is better, with a maximum value of 1.0) increases with increasing labels. The S^3^LDS outperforms the TCN across all labeled data amounts (Fig. 3D, solid lines) using these position features, demonstrating how the addition of unlabeled frames can improve supervised classification performance.

While modeling the raw pose estimates is intuitive, the SLDS framework can accommodate any type of behavioral features, and has been used, for example, with depth cameras (Wiltschko et al. 2015; Johnson et al. 2016; Markowitz et al. 2018; Wiltschko et al. 2020; Costacurta et al. 2022; Markowitz et al. 2023), latent representations from autoencoders (Batty et al. 2019; Whiteway et al. 2021), and other behavioral features (Buchanan et al. 2017). While our goal here is not to determine the optimal behavioral representation for our models, we find that simply concatenating the pose vector $x_{t}$ with its velocity $x_{t}-x_{t-1}$ leads to a new behavioral representation ${\tilde{x}}_{t}=\left[{\text{x}}_{t};x_{t}-{\text{x}}_{t-1}\right]$ of position-velocity features which outperforms the position features $x_{t}$ in both model types (Fig. 3D, dashed lines). With this new behavioral representation, the performance improvements due to unlabeled frames disappear.

To further investigate this, we next look at a snippet of model predictions using ${\tilde{x}}_{t}$ (Fig. 3C). One observation is that S^3^LDS makes mistakes across a wider range of behavior classes. We compute the confusion matrices for each model across all test videos, but do not see any obvious structural differences between the models (Fig. 3E). However, another observation is that the S^3^LDS predictions contain some rapid state switches. One cause of these rapid switches would be two or more classes whose probabilities are similar, and small amounts of noise can lead to switches between these states. To test this hypothesis, we computed the entropy of the discrete state probabilities for each action class. The models are evenly matched for the false positives (Fig. 3F). However, for true positives the entropy for the S^3^LDS is higher across all classes, indicating the semi-supervised model is not as confident as the supervised one.

**Figure 4:**
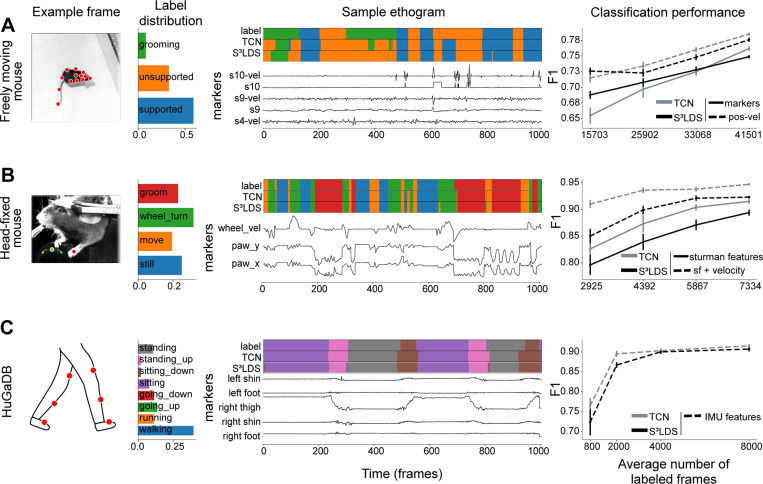
Supervised vs semi-supervised results across datasets. Conventions as in Fig. 3. As in the head-fixed fly, we find that using position-velocity features improves performance over the position features across both model types, and in all datasets the TCN performs best. **A:** Results on the freely moving mouse dataset. Rather than using the raw markers, we compute the features introduced in Sturman et al. 2020. These features compute transformations on the raw markers, including distances and angles between different groups of keypoints. **B:** Results on the head-fixed mouse dataset. **C:** Results on the HuGaDB dataset. The data is collected from sensors that already contain velocity data, so we only use one set of features.

We find similar patterns in the remaining datasets: the S^3^LDS can outperform the TCN when using position features (freely moving mouse), but the TCN performs best across all datasets when incorporating temporal information with the position-velocity features (Fig. 4, Fig. S2).

The finding that inclusion of temporal information in the observations leads to improved classification performance raises a related question: are the temporal components of the S^3^LDS necessary for accurate action segmentation? To answer this question we perform an ablation study. We start with the semi-supervised Gaussian Mixture Deep Generative Model (GMDGM) (Willetts et al. 2020), a static analogue of the S^3^LDS. The GMDGM was developed to model static data such as images, and as such neither the approximate posteriors nor the generative model take temporal information into account. As in the S^3^LDS, we allow for some of the discrete states to be observed (Fig. S3A). To bridge the gap between the GMDGM and the S^3^LDS we introduce the GMDGM-TCN, which uses the static generative model of the GMDGM and the temporal context-aware approximate posterior of the S^3^LDS (Fig. S3B). The GMDGM and GMDGM-TCN thus highlight the effects of ablating different aspects of the dynamic structure in the S^3^LDS.

Across all datasets, we find the GMDGM performs much worse than the temporal models when the observations do not contain temporal information (Fig. S4). Except for the fly dataset, the GMDGM performance is improved upon by adding temporal information into the inference network (the GMDGM-TCN). Interestingly, adding temporal information to the generative model (the S^3^LDS) does not meaningfully improve performance across any of the datasets, regardless of the behavioral features used. We conclude that incorporating temporal information in the observations and the approximate posterior provide complementary improvements in model performance, and incorporating temporal information into the generative model does not provide additional gains.

Finally, we look at the role of nonlinearities in the S^3^LDS. In our implementation we use linear transformations for the recurrent state transitions (Eq. 4) and the continuous latent space dynamics (Eq. 2). We also implement a semi-supervised *non*linear dynamical system (S^3^NLDS) where we replace each of these linear transformations with a one-hidden-layer dense neural network (Methods). The flexibility of our amortized variational inference scheme allows for such changes to the generative model with no corresponding changes in the inference. We find that – at least for these datasets – there is no benefit to using nonlinearities, regardless of the behavioral representation (Fig. S5).

### 2.4 Adding sparse labels to an unsupervised method modifies the latent space

We now turn to comparisons between the S^3^LDS and a closely related unsupervised model, keypoint-MoSeq (Weinreb et al. 2024). Keypoint-Moseq is also in the class of SLDS models, but differs in several key aspects. Importantly, keypoint-MoSeq uses a different prior on the discrete states that allows the model to choose the number of states from the data; this also requires a very different inference approach than the one developed for the S^3^LDS. Despite these differences (among others), we are interested in comparing the discrete states uncovered by these models.

**Figure 5:**
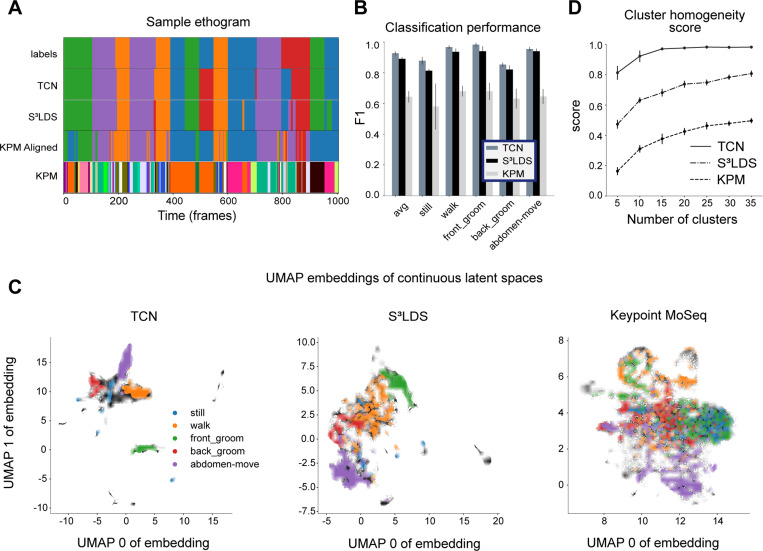
Supervised and semi-supervised latent spaces more closely align with labels than unsupervised latents. All models use position-velocity features and all available training videos for the head-fixed fly dataset. **A:** The top row shows a segment of ground truth labels. The following two rows show predictions from the TCN and S^3^LDS models. The third row shows the state outputs of keypoint-MoSeq (KPM), aligned to the ground truth class with highest overlap on the training data. The final row shows the raw state outputs of keypoint-MoSeq. **B:** F1 scores for the TCN, S^3^LDS and KPM models. Error bars represent the standard deviation of the F1 scores over five trained models (different initialization seeds). **C:** 2D UMAP embedding of continuous latents colored by discrete labels for three different models. **D:** The addition of hand labels produces more homogeneous clusters in the models’ latent spaces. Error bars represent the standard deviation of the cluster scores over five models. We use a range of cluster numbers to show that cluster scores are not biased by cluster size.

We fit keypoint-Moseq on the position-velocity fly features, and find 37 discrete states. We then match each of these states to the labeled action class that contains the most overlap in the training data, and find good overlap with the ground truth labels on held-out test data (Fig. 5A). We quantify this overlap with the same F1 score as before, and find that keypoint-MoSeq achieves competitive results, but unsurprisingly the TCN and S^3^LDS – which both have access to the hand labels during training – perform better on this supervised metric (Fig. 5B).

**Figure 6:**
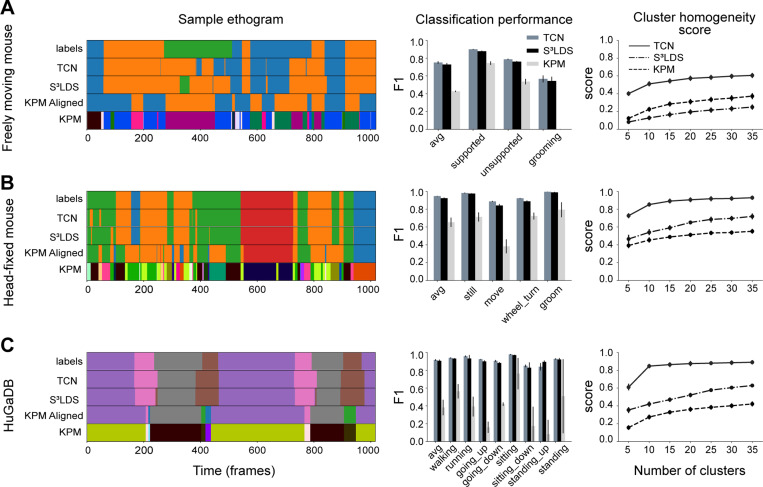
Keypoint-MoSeq performance on non-fly datasets: position-velocity features. Models are trained with position-velocity features for all of the datasets. The mice datasets (panels A and B) use position and velocity information constructed from the pose estimates, the HuGaDB dataset uses inertial sensor data (panel C). Other conventions as in Fig. 5. As in the fly dataset, we find the TCN, which is purely supervised, achieves the highest alignment of the latent space with the ground truth labels as measured by the cluster homogeneity score.

It is also possible to investigate the effects of hand labels on the continuous latent space of each model type. The latent embeddings are computed by passing the observations through the trained encoders for the TCN and S^3^LDS, and performing inference of the continuous latents in keypoint-MoSeq. We first project the latents of each model in 2D space using UMAP (McInnes et al. 2018) (Fig. 5C). The points with corresponding hand labels are colored, revealing that similar behaviors are clustered together across all model types. To quantify this observation, we next perform k-means clustering in the original latent space for each model. We then compute a cluster homogeneity score (Rosenberg et al. 2007) that measures the extent to which the k-means clusters contain data points from a single behavior class. The S^3^LDS achieves a higher score than keypoint-MoSeq, demonstrating that the presence of hand labels during training does in fact shift the continuous latent space to be more aligned with the labeled behavior classes (Fig. 5D). The TCN, which is purely supervised, scores much higher than the other two models since its only objective is to properly separate the hand labels. These observations are replicated across the other datasets (Fig. 6) and with various other cluster quality metrics (Fig. S6). We show results on the non-temporal features in the supplemental material (Figs. S7, S8, S9).

We close by noting the F1 score and cluster quality metrics are all based on hand labels; a purely unsupervised method like keypoint-MoSeq is not trained to optimize these types of metrics. Rather, keypoint-MoSeq (and related methods) finds action classes defined by statistical regularities in the behavioral dynamics. Our finding here is that these statistical regularities may not match up to human-defined behavioral classes as precisely as models trained specifically for this task.

## 3 Discussion

We surveyed a range of animal action segmentation models spanning supervised, semi-supervised, and unsupervised learning paradigms. Across four behavioral datasets, we established that supervised TCN models trained with both static and dynamic pose information performed the best on supervised classification metrics. We also proposed the S^3^LDS, a semi-supervised method for animal action segmentation that incorporates a small amount of discrete labels. With the S^3^LDS we were able to selectively ablate different aspects of the model and assess how they impacted performance; we found that including temporal information in the inference network and the observations is critical, but temporal information in the generative model did not lead to performance improvements. Furthermore, we compared these models to the unsupervised keypoint-MoSeq, and showed how the inclusion of labeled data during training shapes the latent spaces of these models to be more aligned with labeled classes.

A straightforward but important conclusion from our work is that the behavioral representation used for action segmentation – whether supervised, unsupervised, or something in between – plays a crucial role in the performance of the algorithm. Sturman et al. 2020 did not use raw pose estimation outputs for action segmentation, but rather computed a set of derived quantities like distances and angles between different keypoints. We confirmed that this representation is much more performant than the raw keypoints themselves (data not shown), but also found that additionally using the velocities of these features led to further improvements. But what are the “best” set of features for any given dataset? The problem of featurization becomes even more complex in the case of social interactions; for example, both the MARS (Segalin et al. 2021) and SimBA (Goodwin et al. 2024) packages compute hundreds of behavioral features within and between animals as input to their classifiers. There has been some work on automating this process. Sun et al. 2020 use large amounts of unlabeled data to build an effective feature extractor from pose data, and then use simple, fully supervised classifiers for the final stage. Similarly, Bohnslav et al. 2021 use a set of unsupervised feature extractors to bypass pose estimation and directly compute abstract behavioral representations from video frames, which are then fed into fully supervised classifiers.

An important direction for future work is to leverage the S^3^LDS framework for semi-*un*supervised learning (Willetts et al. 2020; Davidson et al. 2021); that is, where we provide a small number of labels for some behavioral categories, but also provide the model with capacity to discover completely novel classes simultaneously. This scenario allows experimenters to precisely quantify known behaviors of interest while also capturing previously unknown behaviors in the data. Another avenue for future work involves modeling the social interactions between multiple animals. Recent work has modeled the interactions between multiple brain regions using rSLDS-like models (Glaser et al. 2020; Karniol-Tambour et al. 2024), and the flexibility of our approach – as well as the relative ease with which we can label discrete animal behaviors compared to discrete brain states – make the S^3^LDS an interesting starting point for such modeling efforts.

## 4 Methods

### 4.1 Datasets

#### Head-fixed fly.

A head-fixed fly engaged in spontaneous behaviors on an air-supported ball (Schaffer et al. 2023). A camera captured a side view of the fly at 70 Hz. We tracked eight points across the legs and abdomen using five supervised Lightning Pose networks, followed by Ensemble Kalman Smoothing (Biderman et al. 2024).

We consider five behavior categories: still, walk, front groom, back groom, and abdomen move. We labeled behaviors in chunks of 50 to several hundred contiguous time points using the DeepEthogram GUI (Bohnslav et al. 2021). Flies often engage in behaviors for longer than 50 frames, so the selected chunks did not contain any transitions from one behavior to another. We use five labeled videos for training, and five for testing. The dataset contains 1.01M frames, only 16.6k of which are labeled (∼1.6%; Table S1). We train the models on 2, 3, 4, and 5 of the training videos to see how our models perform with different amounts of labeled data. For the models trained with 5 videos, we run each model five times with different random seeds. For the other models, we subsample different permutations of the training videos, i.e. for 2 videos, we take 5 random permutations of 2 videos from the list of all 5 training videos.

#### Freely moving mouse.

In this publicly available dataset, a mouse freely moved around an open arena (Sturman et al. 2020). A camera captured a top-down view of the mouse at 25 Hz. Thirteen points were tracked across the tail, body, and head of the mouse. Instead of using the raw pose data for observations, we use a set of 22 features computed from the pose data based on distances, angles, and areas, following Sturman et al. 2020. This provides a position- and orientation-invariant representation that we did not need to account for in the head-fixed fly dataset.

We consider three behavior categories: unsupported rearing, supported rearing (when the mouse uses the wall of the arena), and grooming. In the rest of the frames, there is no specific action or behavior displayed, so we do not train any of the models to identify this “other” category. We only consider the three specific behaviors described above in our analyses. We use 10 labeled videos for training, and 10 for testing. The dataset contains 280k frames, 83k of which are labeled (∼29.5%; Table S2). We train our models on 4, 6, 8, and 10 videos. We choose the permutations of these videos similar to the head-fixed fly videos.

#### Head-fixed mouse.

This is a publicly available dataset from the International Brain Lab (IBL et al. 2023). A head-fixed mouse performed a perceptual decision-making task, using a wheel to indicate its decision (IBL et al. 2021; IBL et al. 2023). Two cameras – “left” (60 Hz) and “right” (150 Hz) – capture roughly orthogonal side views of the mouse’s face and upper trunk during each session (IBL et al. 2022), though we only use the left view. Multiple points are tracked across the paws and face, though for this work we only consider a single tracked point on the paw closest to the camera. In addition to the paw coordinates, we also use the 1D wheel velocity as input to the models. See example videos from one of the sessions here: https://viz.internationalbrainlab.org/app?dset=bwm&pid=94fcff55-2da2-4366-a2c7-2f58c05b54dc&tid=57&cid=598&qc=0#trialviewer.

We consider four behavioral categories for this single paw: still, moving without turning the wheel, moving while turning the wheel, and grooming. We labeled behaviors using the DeepEthogram GUI (Bohnslav et al. 2021). We use five labeled videos for training, and five for testing. The dataset contains 1M frames, 14k of which are labeled (∼1.4%; Table S3). We train the models on 2, 3, 4, and 5 of the training videos, as described for the other datasets.

#### Human Gait Database.

The Human Gait Database (HuGaDB) is a publicly available action segmentation dataset that contains lower limb activities such as walking, running, and sitting for a total of 18 subjects (Chereshnev et al. 2017). HuGaDB measured movements from six inertial measurement units (IMUs) with a 60 Hz sampling frequency. The IMUs were placed on each individual’s right and left feet, shins, and thighs. Each inertial sensor tracks the acceleration data and gyroscope data on each of the three x,y,z axes.

We consider eight behavioral categories: walking, running, going up, going down, sitting, sitting down, standing up, and standing. The original dataset contained four additional behaviors that we excluded from our analysis. Two of those behaviors, “bicycling” and “sitting in car,” were not present in the publicly available dataset. The IMU data for the classes “up by elevator” and “down by elevator” did not seem to contain measurable variability. The dataset contains 517k frames, all of which are labeled (Table S4). We train our model on 100, 250, 500, and 1000 labeled frames from each class. For each number of training frames, we randomly select the specified number of labeled frames, and remove the rest of the labels for training.

### 4.2 Semi-supervised linear dynamical system (S^3^LDS)

#### 4.2.1 S^3^LDS model formulation

Our starting point is the switching linear dynamical system (SLDS) defined in Eqs. 1–4. The specific model that we implement further generalizes the above model by allowing the transitions between discrete states to be recurrent, and the observation mapping to be nonlinear, leading to a modified recurrent switching linear dynamical system (rSLDS) model (Dong et al. 2020; Karniol-Tambour et al. 2024):

(5) $$p_{\theta }\left(x_{1:T},y_{1:T},z_{1:T}\right)=p_{\theta }\left(x_{1}|z_{1}\right)p_{\theta }\left(z_{1}\right)p\left(y_{1}\right)\prod_{t=2}^{T}p_{\theta }\left(x_{t}|z_{t}\right)p_{\theta }\left(z_{t}|z_{t-1},y_{t}\right)p_{\theta }\left(y_{t}|y_{t-1},z_{t-1}\right)$$

(6) $$p_{\theta }\left({\text{x}}_{t}|z_{t}\right)=N\left({\text{x}}_{t}|g_{\theta }\left(z_{t}\right),S\right)$$

(7) $$p_{\theta }\left(z_{t}|z_{t-1},y_{t}\right)=N\left(z_{t}|A_{y_{t}}z_{t-1}+b_{y_{t}},Q_{y_{t}}\right)$$

(8) $$p_{\theta }\left(y_{t}|y_{t-1},z_{t-1}\right)=\text{Cat}\left(y_{t}|\text{Softmax}\left(R_{y_{t-1}}z_{t-1}+r_{y_{t-1}}\right)\right)$$

(9) $$p\left(z_{1}\right)=N\left(z_{1}|0,I\right)$$

(10) $$p\left(y_{1}\right)=Cat(y_{1}|\pi ),$$

where $R_{y_{t}}$ and $r_{y_{t}}$ define the recurrent transition associated with state $y_{t}$, and $g_{\theta }$ is a one-hidden-layer dense neural network that is shared across all discrete states. For our investigation of nonlinearities (Fig. S5), we replaced each of the linear transformations in Eqs. 7 and 8 with a one-hidden-layer dense neural network.

#### 4.2.2 S^3^LDS inference and learning

Switching models contain discrete latent variables, and therefore we cannot simply use the reparameterization trick commonly employed in variational inference schemes. One option is to use a continuous relaxation of the discrete variables such as the Gumbel Softmax (Jang et al. 2016), Concrete (Maddison et al. 2016), or REBAR (Tucker et al. 2017). However, in practice this approach has not been successful with switching *non*linear models (SNLDS) (Dong et al. 2020). Both Dong et al. 2020; Karniol-Tambour et al. 2024 use the exact posterior of the discrete states under the generative model, and perform inference using the forward-backward algorithm. We take a different approach and marginalize out the discrete state such that we obtain a soft mixture of dynamics at each time step, similar to the Kalman VAE (Fraccaro et al. 2017).

We define the approximate posteriors for this model in a way that leads to a classifier $q_{\phi }\left(y_{t}|{\text{x}}_{1:T}\right)$, which is trained on both labeled and unlabeled data (Willetts et al. 2020). We also incorporate temporal information in our approximate posteriors to more closely match the generative model (Krishnan et al. 2015; Krishnan et al. 2017). We propose to do so with a temporal convolution network (Lea et al. 2016; Lea et al. 2017). To begin, let us assume we have no observed labels $y_{t}$; we define a joint posterior over $\left\{y_{1:T},z_{1:T}\right\}$ that factorizes over time:

(11) $$q_{\phi }\left(y_{1:T},z_{1:T}|x_{1:T}\right)=\prod_{t=1}^{T}q_{\phi }\left(y_{t},z_{t}|x_{T_{t}}\right)$$

where $T_{t}$ defines a set of time points, for instance {1,…,T} if we condition on all observations, or {t−τ,…,t+τ} if we condition on a window of observations centered at time t. We further factorize $q_{\phi }\left(y_{t},z_{t}|xT_{t}\right)$ as

(12) $$q_{\phi }\left(y_{t},z_{t}|x_{T_{t}}\right)=q_{\phi }\left(y_{t}|x_{T_{t}}\right)q_{\phi }\left(z_{t}|x_{T_{t}},y_{t}\right)$$

(13) $$q_{\phi }\left(y_{t}|x_{T_{t}}\right)=\text{Cat}\left(y_{t}|\pi_{\phi }\left({\text{x}}_{T_{t}}\right)\right)$$

(14) $$q_{\phi }\left(z_{t}|x_{T_{t}},y_{t}\right)=N\left(z_{t}|\mu_{\phi }\left(x_{T_{t}},y_{t}\right),\text{diag}\left(\sigma_{\phi }^{2}\left(x_{T_{t}},y_{t}\right)\right)\right).$$

This combination of amortization and variational approximation results in the distribution $q_{\phi }\left(y_{t}|x_{T_{t}}\right)$ which can be interpreted as a classifier that can efficiently predict the discrete behavior state after training the model is complete.

There has been much work on developing inference strategies for rSLDS models (Linderman et al. 2016; Linderman et al. 2017; Nassar et al. 2018). Our contribution here is the development of an efficient and flexible amortized variational inference strategy that incorporates both labeled and unlabeled data. We note that the proposed inference strategy leads to the definition of an approximate posterior $q_{\phi }\left(y_{t}|x_{T_{t}}\right)$. This approximate posterior – which is trained on both labeled and unlabeled data – also serves as a classifier, predicting the discrete action class $y_{t}$ from the observed data $x_{1:T}$ using a neural network (Willetts et al. 2020). Existing inference methods for rSLDS models do not use labeled data or an amortized inference network (Linderman et al. 2016; Linderman et al. 2017; Nassar et al. 2018). We use a TCN (Lea et al. 2016; Lea et al. 2017) as our model backbone, as motivated in a previous section. This inference approach allows us to directly compare classifiers that are trained with labeled data only (i.e., supervised) or with both labeled and unlabeled data (i.e., semi-supervised).

##### ELBO with all labels observed.

Let us first consider the variational lower bound assuming all $y_{t}$ are observed (details in Appendix A). We will use ˜ to denote that a variable is sampled from its approximate posterior using the reparameterization trick (Kingma et al. 2013):

(15) $$L_{l}\left({\text{x}}_{1:T},y_{1:T}\right)=\sum_{t=1}^{T}\log p\left({\text{x}}_{t}|{\tilde{z}}_{t}\right)\;\;-\text{KL}\left[q\left(z_{1}|x_{T_{1}},y_{1}\right)\|p\left(z_{1}\right)\right]+\log p\left(y_{1}\right)\;\;-\sum_{t=2}^{T}KL\left[q\left(z_{t}|x_{T_{t}},y_{t}\right)||p\left(z_{t}|{\tilde{z}}_{t-1},y_{t}\right)\right]\;\;+\sum_{t=2}^{T}\log p\left(y_{t}|y_{t-1},{\tilde{z}}_{t-1}\right).$$

Note that Eq. 15 does not explicitly contain a term that looks like a classifier; we will address this in a later section.

##### ELBO with all labels unobserved.

Next, let us derive the variational lower bound assuming all $y_{t}$ are unobserved (details in Appendix A).

(16) $$L_{u}\left({\text{x}}_{1:T}\right)=\sum_{t=1}^{T}\sum_{k=1}^{K}\left(q\left(y_{t}=k|x_{\tau_{t}}\right)\log p\left({\text{x}}_{t}|{\tilde{z}}_{t}^{k}\right)\right)\;\;-\sum_{k=1}^{K}\left(q\left(y_{1}=k|x_{T_{t}}\right)\text{KL}\left[q\left(z_{1}|x_{T_{1}},y_{1}=k\right)∥p\left(z_{1}\right)\right]\right)-\text{KL}\left[q\left(y_{1}|x_{T_{1}}\right)∥p\left(y_{1}\right)\right]\;\;-\sum_{t=2}^{T}\sum_{k=1}^{K}\sum_{k^{\prime }=1}^{K}(q\left(y_{t}=k|x_{T_{t}}\right)q\left(y_{t-1}=k^{\prime }|x_{T_{t-1}}\right)\;\;\text{KL}\left[q\left(z_{t}|x_{T_{t}},y_{t}=k\right)∥p\left(z_{t}|{\tilde{z}}_{t-1}^{k^{\prime }},y_{t}=k\right)\right])\;\;-\sum_{t=2}^{T}\sum_{k=1}^{K}\left(q\left(y_{t-1}=k|x_{T_{t-1}}\right)\text{KL}\left[q\left(y_{t}|x_{T_{t}}\right)∥p\left(y_{t}|y_{t-1}=k,{\tilde{z}}_{t-1}^{k}\right)\right]\right)$$

where ${\tilde{z}}_{t}^{k}∼q\left(z_{t}|xT_{t},y_{t}=k\right)$ and ${\tilde{z}}_{t-1}^{k}∼q\left(z_{t-1}|x_{T_{t-1}},y_{t-1}=k\right)$. A nice property of this lower bound is that each of the individual terms in the unlabeled ELBO (Eq. 16) reduces to the corresponding term in the labeled ELBO (Eq. 15) when the output of $q\left(y_{t}|x_{T_{t}}\right)$ is a one-hot vector (i.e. observed), which we exploit when computing the full semi-supervised ELBO (more below).

##### Classification loss.

Note that Eq. 16 contains terms of the form $q\left(y_{t}|x_{T_{t}}\right)$, which maps observations to the discrete state. This part of the approximate posterior is a classifier that only appears in the unlabeled ELBO, and hence does not utilize labeled data for learning. Therefore, we can improve classification performance by including a cross entropy loss term for our model to learn the classes. We weight this loss with a hyperparameter α, chosen through cross-validation.

##### Total loss function.

The total loss function for the general semi-supervised case will be a combination of Eqs. 15 and 16 depending on which subset of time points include observed discrete labels. In practice we compute $q\left(y_{t}|x_{T_{t}}\right)$ for all time points, then replace the probability vector with a one-hot vector when the discrete label is observed, and then compute Eq. 16. In addition, we compute the classification loss for all observed discrete labels. See Appendix A for full details.

### 4.3 Gaussian mixture deep generative model (GMDGM)

For one of our baselines we implement the Gaussian mixture deep generative model (GMDGM) proposed in Willetts et al. 2020, which is similar to the S^3^LDS but without temporal dependencies (Fig. S3A). This is a modified version of the semi-supervised deep generative model proposed in Kingma et al. 2014a.

#### 4.3.1 GMDGM model formulation

The generative model is defined as

(17) $$p_{\theta }(x,y,z)=p_{\theta }(x|z)p_{\theta }(z|y)p(y)$$

(18) $$p_{\theta }(\text{x}|z)=N\left(\text{x}|g_{\theta }(z),R\right)$$

(19) $$p_{\theta }(z|y)=N\left(z|f_{\theta }(y),\text{diag}\left(\sigma_{\theta }^{2}(y)\right)\right)$$

(20) p(y)=Cat(y∣π).

This model defines an explicit clustering mechanism by conditioning the latents z on the discrete label y , and defining a categorical prior over y . Therefore, even in the absence of labeled data, the GMDGM will attempt to cluster the data.

#### 4.3.2 GMDGM inference and learning

Like the S^3^LDS we perform approximate inference in the GMDGM model. Furthermore, we structure the approximate posteriors in a way that leads to a classifier $q_{\phi }(y|x)$, which is trained on both labeled and unlabeled data.


(21) $$q_{\phi }(y,z|x)=q_{\phi }(y|x)q_{\phi }(z|x,y)$$

(22) $$q_{\phi }(y|\text{x})=\text{Cat}\left(y|\pi_{\phi }(\text{x})\right)$$

(23) $$q_{\phi }(\text{z}|\text{x},y)=N(\text{z}|\mu_{\phi }(\text{x},y),\text{diag}\left(\sigma_{\phi }^{2}(\text{x},y)\right)).$$

For the GMDGM-TCN model we use the same approximate posteriors as the S^3^LDS model, defined in Eqs. 12–14 (Fig. S3B).

The variational lower bound for labeled data is

(24) $$L_{l}(x,y)=E_{q(z|x,y)}\left[\log\frac{p(x|z)p(z|y)p(y)}{q(z|x,y)}\right]\;\;=E_{q(z|x,y)}[\log p(x|z)]+\log p(y)-\text{KL}[q(z|x,y)∥p(z|y)]\;\;\approx \log p(x|\tilde{z})+\log p(y)-\text{KL}[q(z|x,y)∥p(z|y)]$$

The variational lower bound for unlabeled data is

(25) $$L_{u}(x)=E_{q(y,z|x)}\left[\log\frac{p(x|z)p(z|y)p(y)}{q(y,z|x)}\right]\;\;=E_{q(y,z|x)}\left[\log\frac{p(x|z)p(z|y)p(y)}{q(y|x)q(z|x,y)}\right]\;\;=\sum_{y}q(y|x)\left[E_{q(z|x,y)}\left[\log\frac{p(x|z)p(z|y)p(y)}{q(y|x)q(z|x,y)}\right]\right]\;\;=\sum_{y}q(y|x)\left[E_{q(z|x,y)}\left[\log\frac{p(x|z)p(z|y)p(y)}{q(z|x,y)}\right]-\log q(y|x)\right]\;\;=\sum_{y}q(y|x)\left[L_{l}(x,y)-\log q(y|x)\right]\;\;=\sum_{y}q(y|x)L_{l}(x,y)-H(q(y|x))\;\;=\sum_{k=1}^{K}q(y=k|x)L_{l}(x,k)-H(q(y|x)).$$

As with the S^3^LDS, note that the term q(y∣x), which we will use as a final classifier, only appears in $L_{u}(x)$ and is therefore not trained with labeled data. We again address this by adding the cross entropy loss between the ground truth label and the distribution provided by q(y∣x) to the lower bound of the labeled data, so that

(26) $$L=E_{x^{\left(l\right)},y^{\left(l\right)}∼D_{l}}\left[L_{l}\left(x^{\left(l\right)},y^{\left(l\right)}\right)+\alpha \log q\left(y^{\left(l\right)}|x^{\left(l\right)}\right)\right]+E_{x^{\left(u\right)}∼D_{u}}\left[L_{u}\left(x^{\left(u\right)}\right)\right],$$

where $D_{l}$ is the labeled dataset and $D_{u}$ is the unlabeled dataset.

### 4.4 Model implementation details

#### 4.4.1 Temporal Convolutional Network backbone

We used the same dilated TCN backbone for the supervised model, GMDGM models, and S^3^LDS. For all models and datasets we used 2 dilation blocks, 4 temporal lags per convolution layer, and 32 filters per convolution layer. Each dilation block consisted of a sequence of 2 sub-blocks (1D convolution layer → leaky ReLU nonlinearity → dropout with probability=0.10), as well as a residual connection between the input and output of the dilation block. The dilation of the convolutional filters starts with 1 for the first dilation block, then increases by a factor of 2 for each additional dilation block. This results in a larger temporal receptive field as the model gets deeper, allowing for learning of longer range dependencies (Yu et al. 2015). For our specific parameters this results in outputs of the network at time t being dependent on time points {t−24,…,t,…,t+24}, or in other words a 0.7 s receptive field for the fly data (70 Hz), a 1.96 s receptive field for the freely moving mouse data (25 Hz), and a 0.82 s receptive field for the head-fixed mouse and human data (both 60 Hz). We experimented with different values of these hyperparameters but found the results to be robust to specific choices.

#### 4.4.2 S^3^LDS model

The S^3^LDS model used two TCN networks as described above: one for the approximate posterior of the discrete states $q\left(y_{t}|x_{T_{t}}\right)$, and one for the approximate posterior of the continuous latents $q\left(z_{t}|x_{T_{t}},{\tilde{y}}_{t}\right)$. We used a one-hidden-layer dense neural network for all the generative model nonlinearities in the S^3^LDS and S^3^NLDS. The number of hidden units was always equal to the dimensionality of the continuous latent space. To determine this, we performed PCA on the observed data and chose the number of dimensions that explained 95% of the variance.

#### 4.4.3 Model training

To train the supervised TCN, GMDGM, and S^3^LDS models we used the Adam optimizer (Kingma et al. 2014b) with a learning rate of 0.0001. Each batch contained 8 sequences of 1000 frames each. We trained the models for 500 epochs. For the semi-supervised models, we tested a range of possible αs (the hyperparameter on the supervised classification loss). We chose α=100 for all of the datasets since that value generalized well across datasets. We anneal the two KL losses and logy (for the labeled data) by linearly increasing the weight from 0 to 1 over 100 epochs.

### 4.5 Tree-based methods

For the tree-based methods we used the same input features as the other models. We used a temporal window of features to match the input to the first layer of the TCN, i.e. the prediction at time t is made from the concatenated behavioral features from {t−4,…,t,…,t+4}.

To train the random forest models we used the function RandomForestClassifier from the sklearn package with the following arguments: n_estimators=6000, max_features=‘sqrt’, criterion=‘entropy’, min_samples_leaf=1, bootstrap=True following Goodwin et al. 2024.

To train the XGBoost models we used the function XGBClassifier from the xgboost package with the following arguments: n_estimators=2000, max_depth=3, learning_rate=0.1, objective=‘multi:softprob’, eval_metric=‘mlogloss’, tree_method=‘hist’, gamma=1, min_child_weight=1, subsample=0.8, colsample_bytree=0.8 following Segalin et al. 2021.

### 4.6 Keypoint-MoSeq

For keypoint-MoSeq, we use both the position features (Fig. S7) as well as the position-velocity features (Figs. 5, 6) as inputs for the head-fixed fly, freely moving mouse, and head-fixed mouse datasets. For the HuGaDB data, we use the IMU features. We used the keypoint_moseq library to train the models. We initialized the model with ARHMM run for fifty iterations followed by training the full keypoint-MoSeq model for 500 additional iterations. We set kappa=1e4 for all models.

## Supplementary Material

Supplement 1

## Data Availability

The pose estimates and discrete state annotations for the freely moving mouse dataset are available at https://github.com/ETHZ-INS/DLCAnalyzer; the raw videos are available at https://zenodo.org/records/3608658.
