# Supplementary material for: A study of animal action segmentation algorithms across supervised, unsupervised, and semi-supervised learning paradigms
Source: ArXiv. 2024 Dec 17:arXiv:2407.16727v2. Originally published 2024 Jul 23. Preprint. [Version 2] (PMC11302674)
Supplement: Supplement 1 [file NIHPP2407.16727v2-supplement-1.pdf]

5 | SUPPLEMENTARY FIGURES

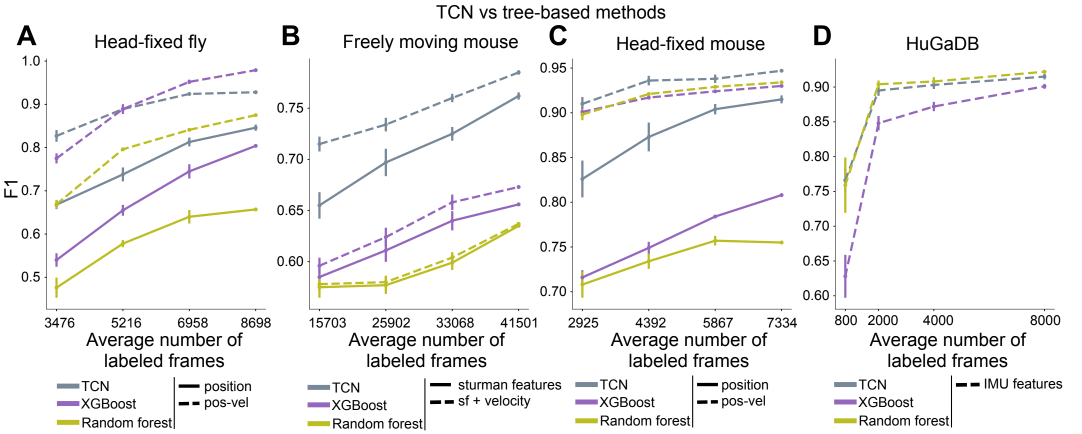

FIGURE S1 **Temporal convolutional networks outperform tree-based methods in supervised classification.** We compare the TCN network to XGBoost and random forest models. We compare models trained on position features (solid lines), as well as on models trained on position-velocity features (dashed lines). For the position feature models, the TCN is the best model on all three datasets that use markers (**A**, **B**, **C**). For the position-velocity features, the TCN is the best model for the freely moving mouse and head-fixed mouse datasets (**B**, **C**). The TCN and XGBoost models perform similarly for the head-fixed fly dataset (**A**), and for the HuGaDB dataset, the TCN and random forest perform similarly (**D**). Given that the TCN performs better or similarly across all four datasets, we select this as the backbone model throughout the rest of the model comparisons in the paper.

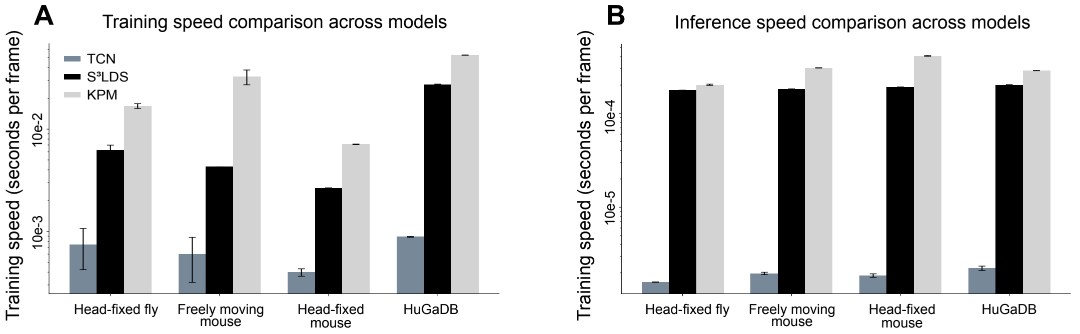

FIGURE S2 Training and inference speed for different model types. For all datasets, the TCN has the fastest training (**A**) and inference (**B**) times, followed by S^{3}LDS, and finally keypoint-MoSeq. Bars represent mean value over 5 random seeds, error bars show s.e.m.

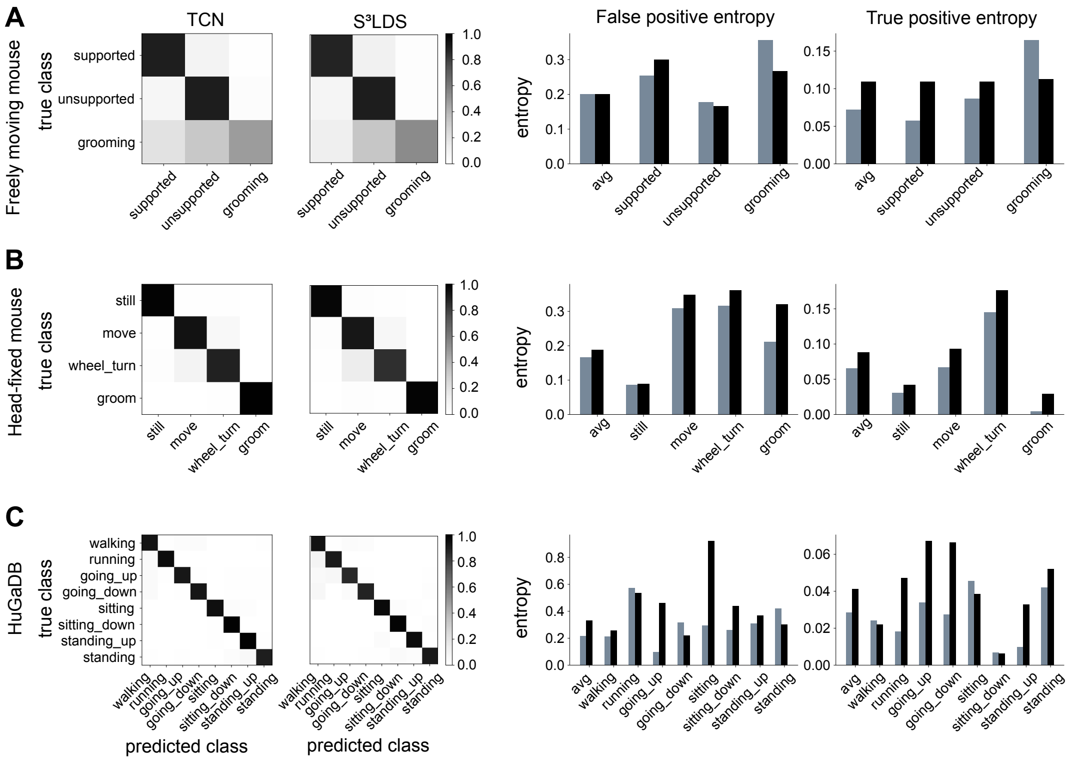

FIGURE S3 Semi-supervised models have less confident predictions than their supervised counterparts. The first two columns show the confusion matrices for the TCN and S^3LDS models, respectively. The latter two columns display the average entropy of the false positives (left) and true positives (right) for both models. In both cases entropies are larger for the S^3LDS model, indicating higher uncertainty in the state distributions. All results are from the models trained on position-velocity features.

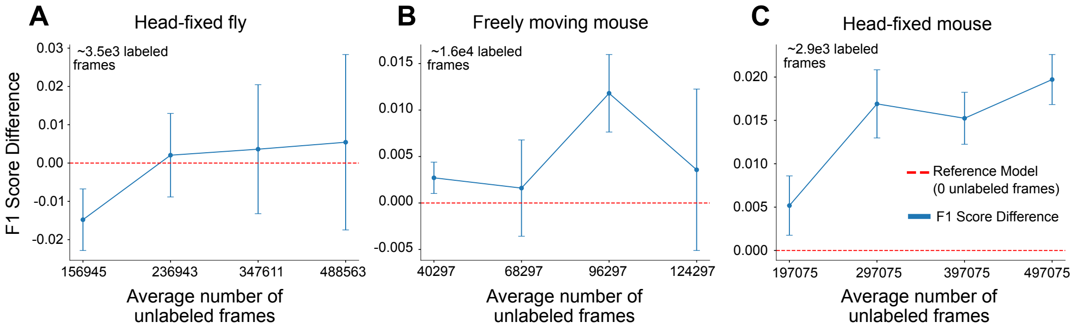

FIGURE S4 Performance of S³LDS when increasing the number of unlabeled frames: position features. F1 score differences comparing a S³LDS trained without any unlabeled frames vs. adding different numbers of unlabeled frames, for models trained with position features. The F1 differences are averaged five models each trained on a different subset of data, with the error bar showing the standard deviation over all five sets of model differences. For all of the datasets, there is a positive trend between the number of unlabeled frames and the corresponding F1 scores. **A**: results on the head-fixed fly dataset. **B**: results on the freely moving mouse dataset. **C**: results on the HuGaDB data.

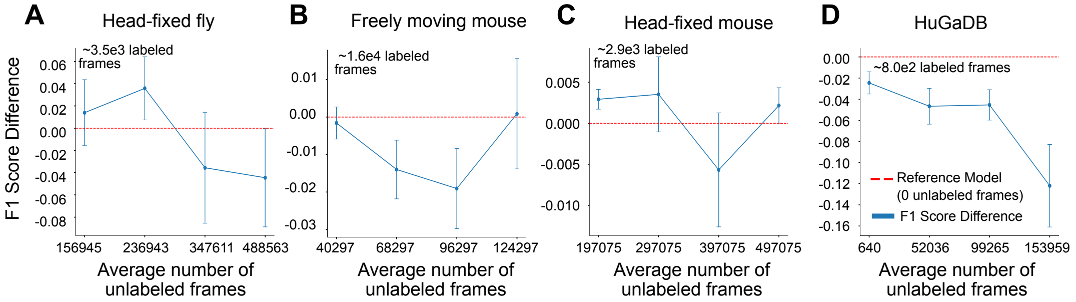

FIGURE S5 Performance of S³LDS when increasing the number of unlabeled frames: position-velocity features. F1 score differences comparing a S³LDS trained without any unlabeled frames vs. adding different numbers of unlabeled frames, for models trained with position-velocity features. For all datasets, there is negative trend between the number of unlabeled frames and the corresponding F1 scores. This suggests that the semi-supervised S³LDS is not a good fit for modeling these more complex features. Conventions as in Fig. S4.

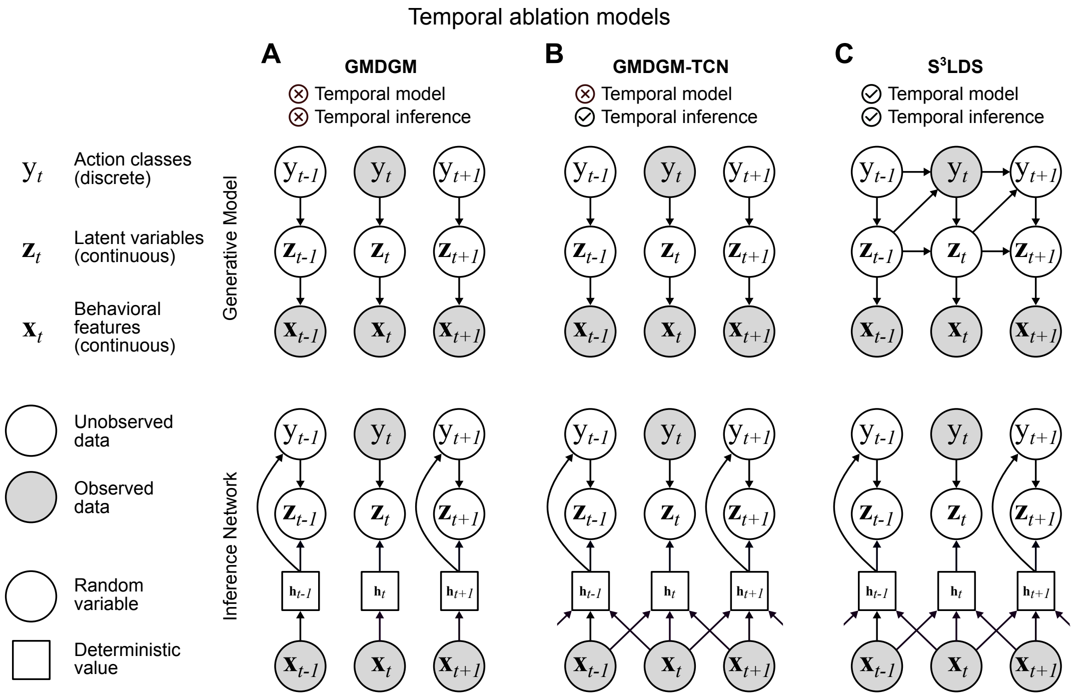

FIGURE S6 S^{3}LDS model ablations. A: Graphical model for the Gaussian Mixture Deep Generative Model (GMDGM) (Willetts et al., 2020). The GMDGM does not contain temporal dependencies in the generative model (top) or the inference network (bottom). **B**: The GMDGM-TCN, which does not contain temporal dependencies in the generative model (top) but does in the inference model (bottom), where we use a window of observed behavioral features for state prediction. **C**: The S^{3}LDS contains temporal dependencies in both the generative model (top) and inference network (bottom). Same schematic as Fig. 2C.

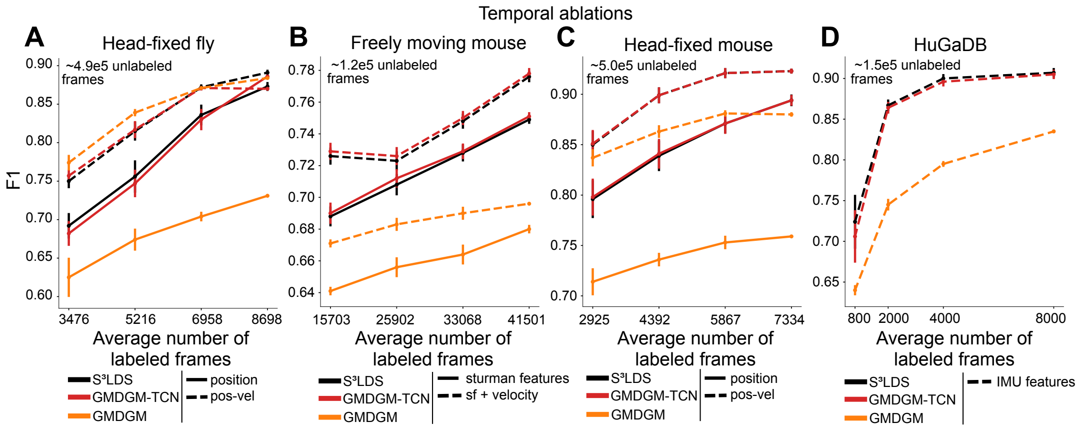

FIGURE S7 The role of temporal information in semi-supervised model performance. Temporal ablations comparing S^{3}LDS (temporal inference and generative model), GMDGM-TCN (temporal inference only), and GMDGM (non-temporal) models. The non-temporal model performs much worse than the other two across all datasets, especially when using non-temporal features.

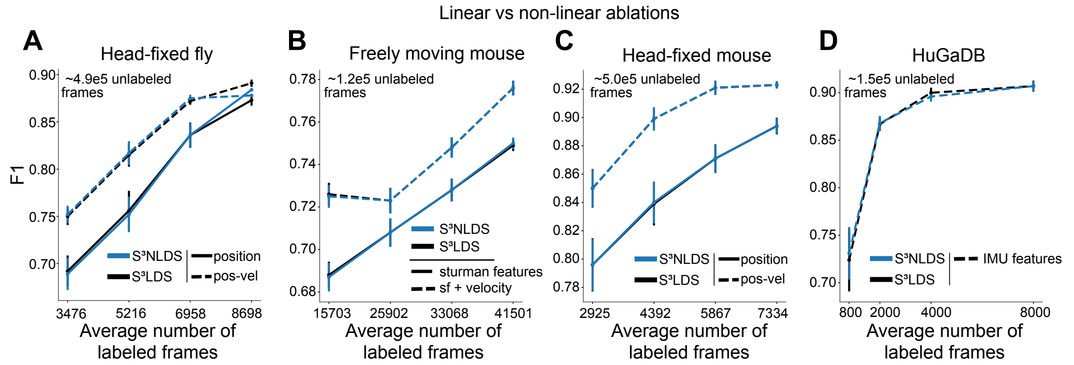

FIGURE S8 The role of nonlinearities in semi-supervised model performance. We compare our S^{3}LDS model to a model that uses one-hidden-layer dense neural networks for the dynamics and recurrent transitions (S^{3}NLDS). The nonlinearities do not improve model performance on any dataset.

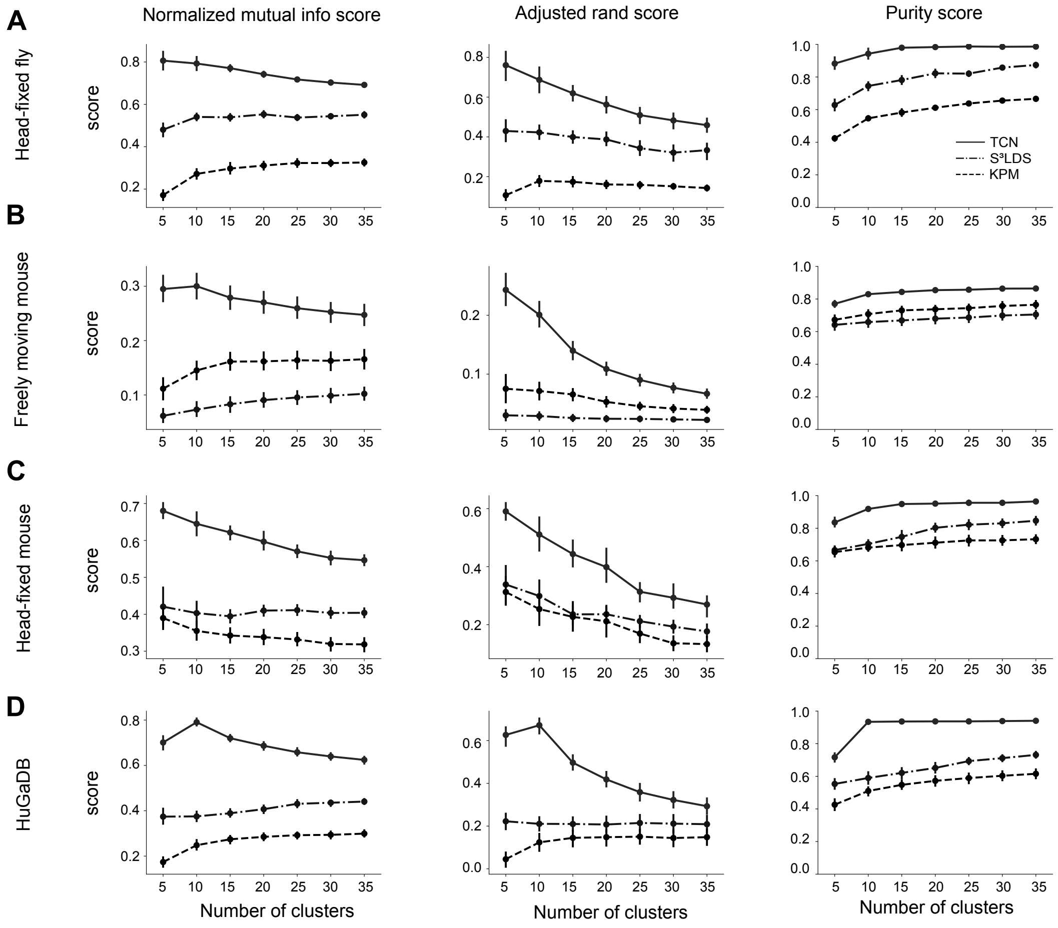

FIGURE S9 The latent spaces of models are shaped by the extent to which labels are used in training: position-velocity features. Cluster metrics for models trained with position-velocity features, which complement the cluster homogeneity score in Figs. 5, 6. The addition of hand labels produces more homogeneous clusters in the models' latent spaces.

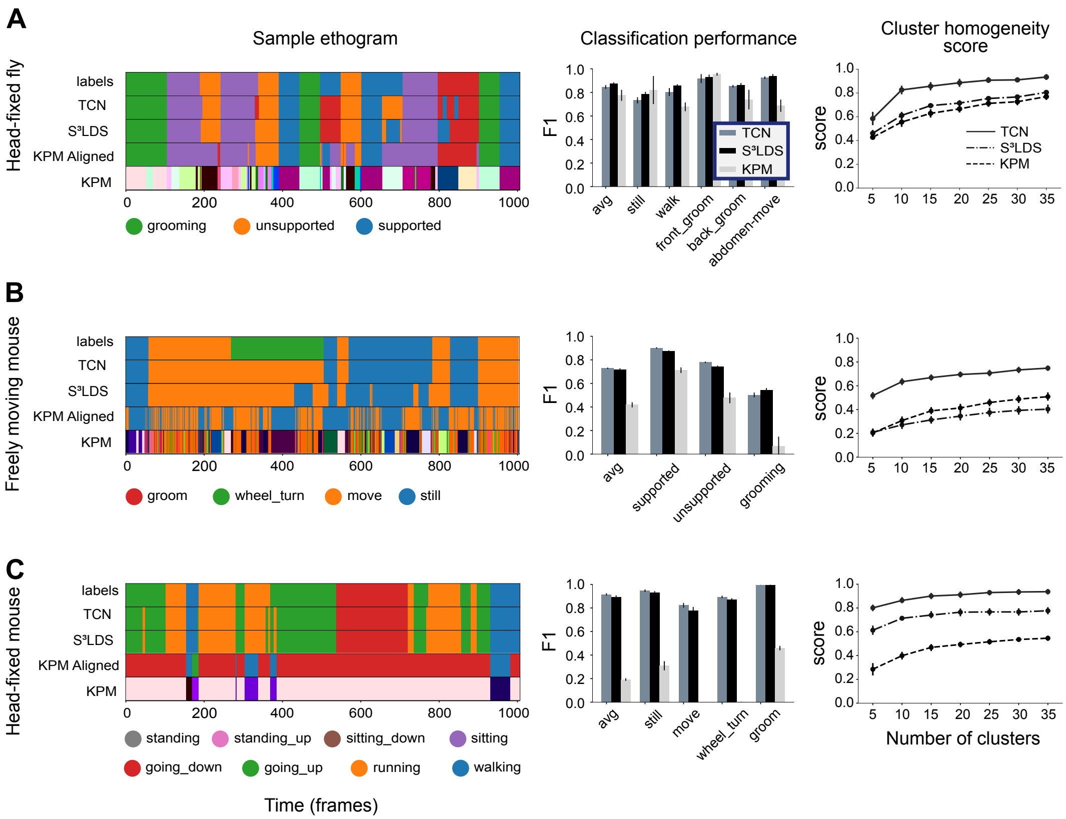

FIGURE S10 Keypoint-MoSeq performance on non-fly datasets: position features. Comparisons between the models using only position features. Conventions as in Fig. 6. We find the TCN, which is purely supervised, achieves the highest alignment of the latent space with the ground truth labels as measured by the cluster homogeneity score.

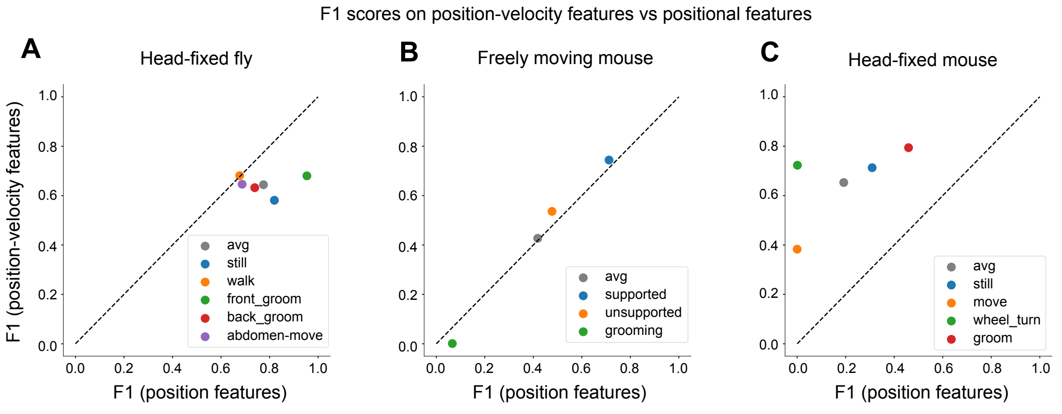

FIGURE S11 Keypoint-MoSeq performance: position-velocity features vs position features. Comparisons between the keypoint-Moseq models trained on position-velocity features vs position features. We find that keypoint-Moseq performs best with the position features on the head-fixed fly dataset, and the position-velocity features on the two mouse datasets.

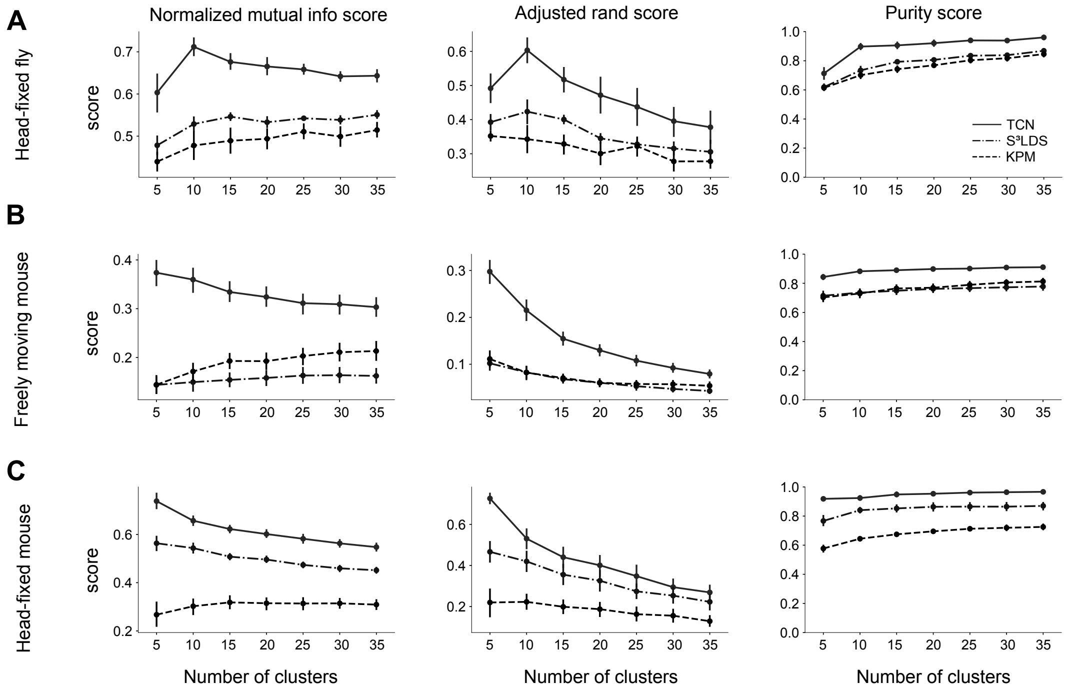

FIGURE S12 Cluster metrics for model latent spaces: position features. Cluster metrics for models trained with position features. Conventions as in Fig. S9.

6 | SUPPLEMENTARY TABLES

	Experiment ID	Total frames	Walk	Still	Front groom	Back groom	Abdomen move
**Train**	2019_08_07_fly2	50000	300	300	100	300	591
	2019_08_08_fly1	94960	300	300	300	150	594
	2019_08_20_fly2	65108	300	300	300	300	1067
	2019_10_10_fly3	141042	300	300	300	300	478
	2019_10_14_fly3	140929	300	300	300	300	318
**Test**	2019_06_26_fly2	45000	300	300	300	300	706
	2019_08_14_fly1	124144	300	300	300	300	693
	2019_08_20_fly3	73055	300	300	300	300	405
	2019_10_14_fly2	139925	300	300	300	300	101
	2019_10_21_fly1	142554	300	300	300	300	0
	**Totals**	1016717	3000	3000	2800	2850	4953

TABLE S1 The number of labeled frames per behavior for the head-fixed fly dataset.

	Experiment ID	Total frames	Supported rear	Unsupported rear	Groom
**Train**	OFT_5	14000	3267	1208	387
	OFT_6	14000	2424	1090	428
	OFT_11	14000	1900	1036	117
	OFT_12	14000	2354	2213	202
	OFT_14	14000	3165	2071	140
	OFT_15	14000	2038	1433	154
	OFT_16	14000	1773	826	493
	OFT_23	14000	1708	263	396
	OFT_24	14000	2130	2312	298
	OFT_38	14000	2564	2598	513
**Test**	OFT_39	14000	2159	2062	570
	OFT_41	14000	2381	1479	178
	OFT_43	14000	3642	338	125
	OFT_44	14000	2314	1069	139
	OFT_49	14000	2638	1120	142
	OFT_50	14000	1550	873	386
	OFT_51	14000	1799	782	442
	OFT_52	14000	2949	1605	547
	OFT_54	14000	2587	2012	367
	OFT_58	14000	2468	1858	577
	**Totals**	280000	47810	28248	6601

TABLE S2 The number of labeled frames per behavior for the freely moving mouse dataset.

	Experiment ID	Total frames	Still	Move	Wheel turn	Groom
**Train**	danlab_DY_009_2020-02-27-001	100000	497	316	374	300
	danlab_DY_018_2020-10-15-001	100000	400	268	241	580
	hoferlab_SWC_061_2020-11-23-001	100000	320	261	494	265
	mrsicflogellab_SWC_058_2020-12-11-001	100000	433	357	358	298
	wittenlab_ibl_witten_26_2021-01-27-002	100000	624	235	353	360
**Test**	churchlandlab_CSHL045_2020-02-27-001	100000	306	368	522	469
	cortexlab_KS020_2020-02-06-001	100000	378	340	322	208
	hoferlab_SWC_043_2020-09-15-001	100000	469	363	306	513
	mrsicflogellab_SWC_052_2020-10-22-001	100000	338	179	494	242
	wittenlab_ibl_witten_27_2021-01-21-001	100000	335	35	536	50
	**Totals**	1000000	4100	2722	4000	3285

TABLE S3 The number of labeled frames per behavior for the IBL head-fixed mouse dataset.

	Experiment ID	Total frames	Walking	Running	Going up	Going down	Sitting	Sitting down	Standing up	Standing
**Train**	subject 05	56199	23034	2154	9820	10246	4215	834	1052	4844
	subject 07	53571	23533	4111	5767	6460	5000	1164	1273	6263
	subject 09	40066	12254	3712	7374	5954	3278	1038	1227	5229
	subject 12	52005	14343	5796	7683	7805	6843	908	1085	7542
	subject 14	55366	23365	5529	8829	6199	4771	920	900	4853
**Test**	subject 06	67747	33386	11334	5367	6214	3153	922	1008	6363
	subject 08	49093	17327	4081	7138	6262	4710	1101	1509	6965
	subject 11	45137	16202	3727	7768	5797	4471	1257	1329	4586
	subject 13	52053	17971	5374	10969	6942	3896	1095	1314	4492
	subject 17	45547	12579	1862	8723	6130	6867	1276	1235	6875
	**Totals**	516784	193994	47680	79438	68009	47204	10515	11932	58012

TABLE S4 The number of labeled frames per behavior for the human gait database.
