# Supplementary material for: A study of animal action segmentation algorithms across supervised, unsupervised, and semi-supervised learning paradigms
Source: ArXiv. 2024 Jul 23:arXiv:2407.16727v1. Preprint. [Version 1] (PMC11302674)
Supplement: Supplementary file 1 [file NIHPP2407.16727v1-supplement-1.pdf]

# 5 Supplementary figures

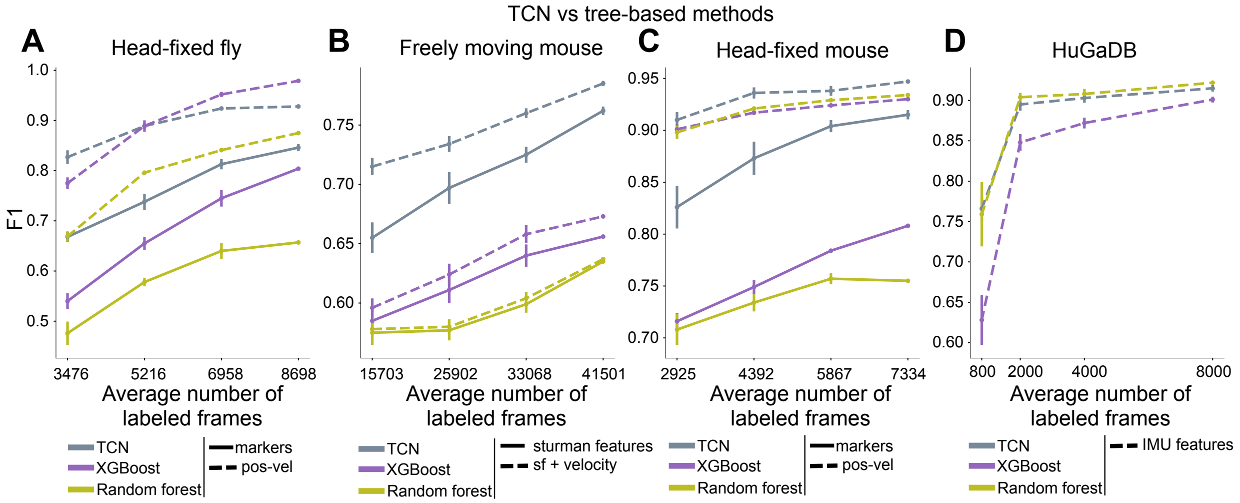


Figure S1: **Temporal convolutional networks outperform tree-based methods in supervised classification.** We compare the TCN network to XGBoost and random forest models. We compare models trained on position features (solid lines), as well as on models trained on position-velocity features (dashed lines). For the position feature models, the TCN is the best model on all three datasets that use markers (**A**, **B**, **C**). For the position-velocity features, the TCN is the best model for the freely moving mouse and head-fixed mouse datasets (**B**, **C**). The TCN and XGBoost models perform similarly for the head-fixed fly dataset (**A**), and for the HuGaDB dataset, the TCN and random forest perform similarly (**D**). Given that the TCN performs better or similarly across all four datasets, we select this as the backbone model throughout the rest of the model comparisons in the paper.

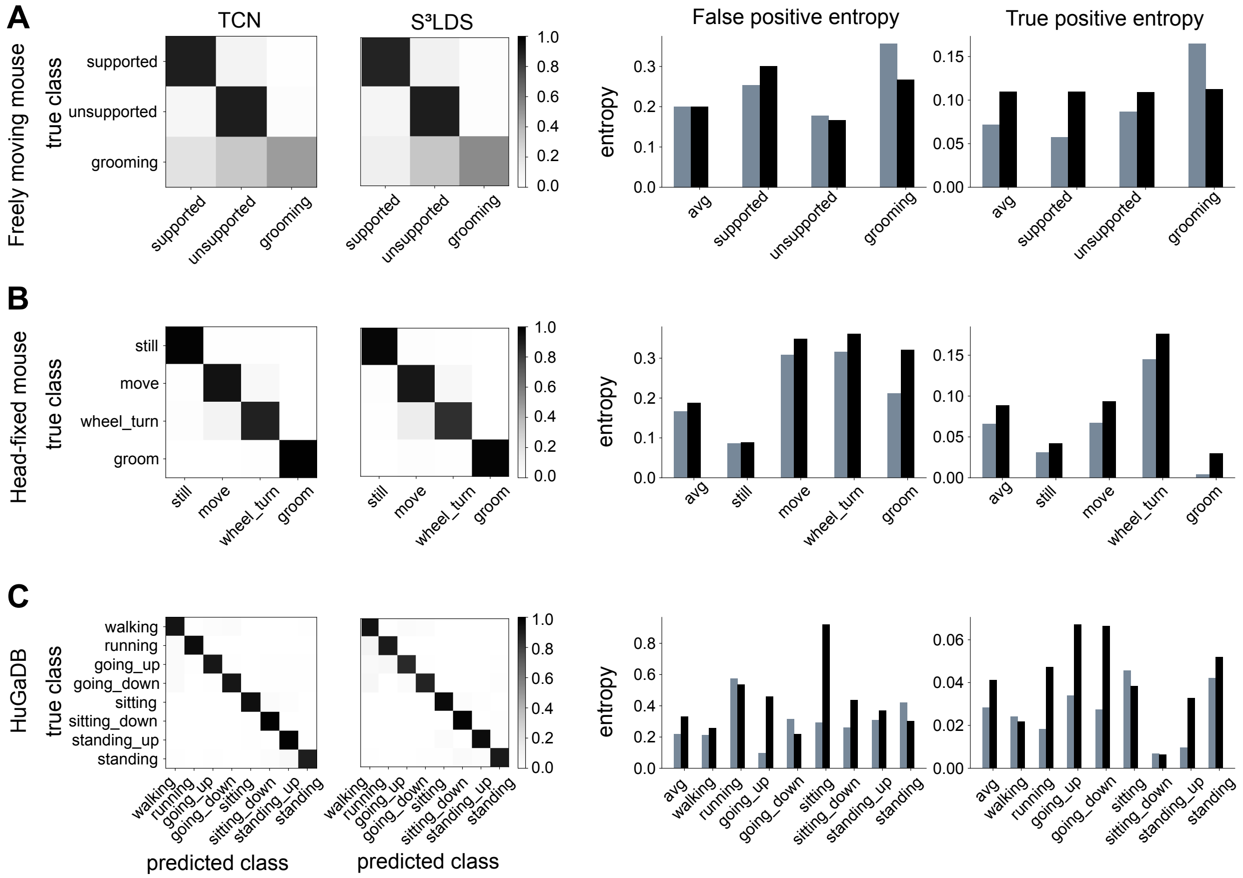


Figure S2: **Semi-supervised models have less confident predictions than their supervised counterparts.** The first two columns show the confusion matrices for the TCN and $S^3$LDS models, respectively. The latter two columns display the average entropy of the false positives (left) and true positives (right) for both models. In both cases entropies are larger for the $S^3$LDS model, indicating higher uncertainty in the state distributions. All results are from the models trained on position-velocity features.

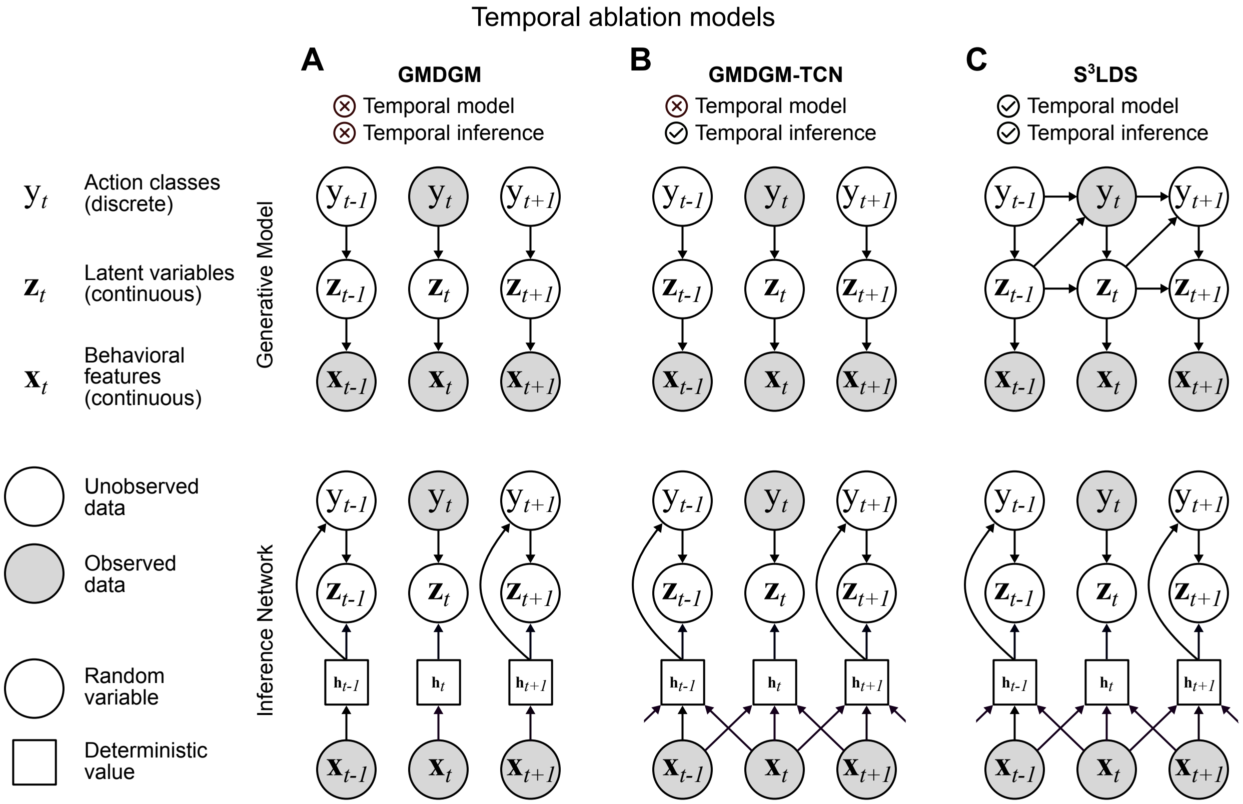


Figure S3: **S$^3$LDS model ablations.** **A**: Graphical model for the Gaussian Mixture Deep Generative Model (GMDGM) (Willetts et al. 2020). The GMDGM does not contain temporal dependencies in the generative model (top) or the inference network (bottom). **B**: The GMDGM-TCN, which does not contain temporal dependencies in the generative model (top) but does in the inference model (bottom), where we use a window of observed behavioral features for state prediction. **C**: The S$^3$LDS contains temporal dependencies in both the generative model (top) and inference network (bottom). Same schematic as Fig. 2C.

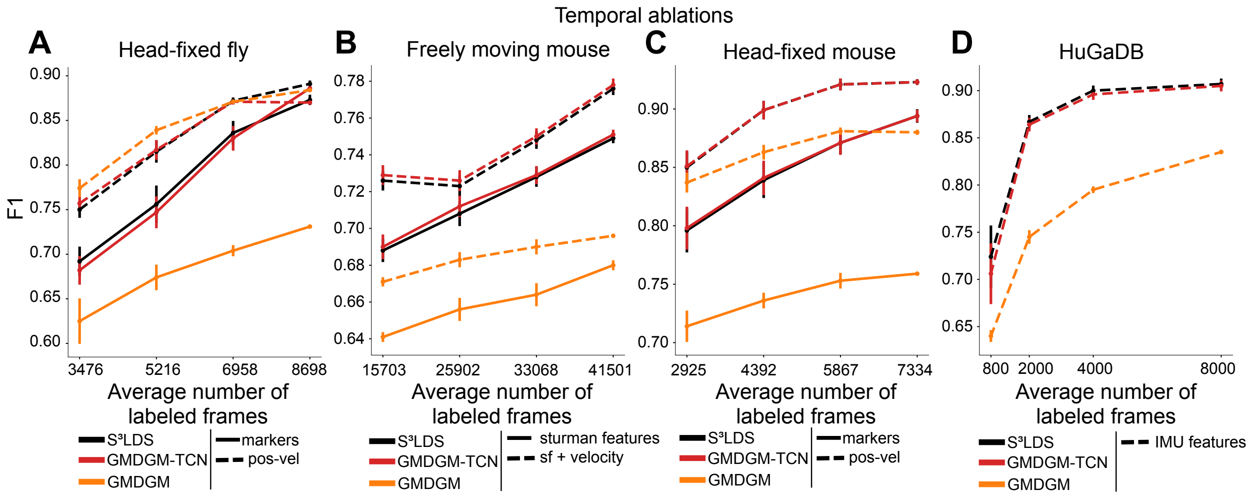


Figure S4: **The role of temporal information in semi-supervised model performance.** Temporal ablations comparing $S^3$LDS (temporal inference and generative model), GMDGM-TCN (temporal inference only), and GMDGM (non-temporal) models. The non-temporal model performs much worse than the other two across all datasets, especially when using non-temporal features.

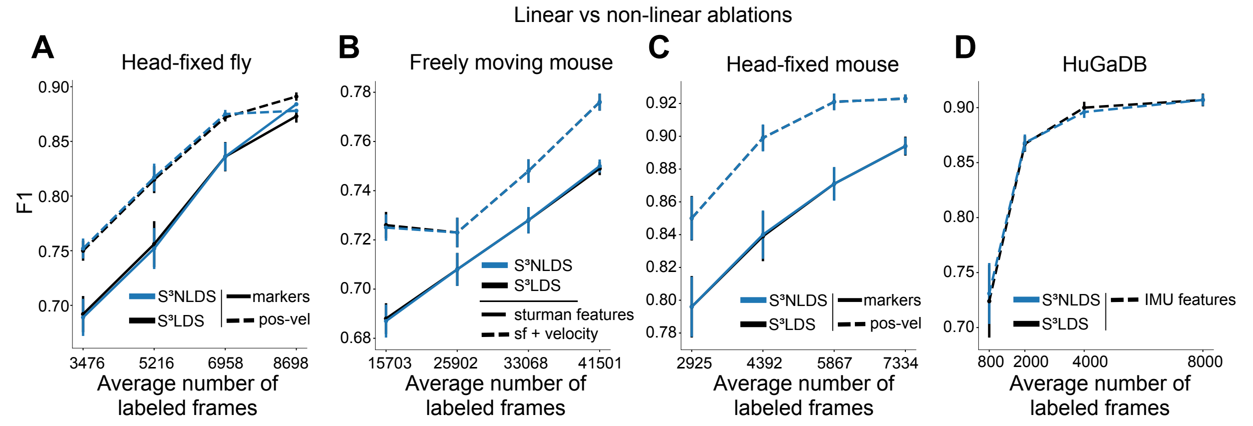


Figure S5: **The role of nonlinearities in semi-supervised model performance.** We compare our $S^3$LDS model to a model that uses one-hidden-layer dense neural networks for the dynamics and recurrent transitions ($S^3$NLDS). The nonlinearities do not improve model performance on any dataset.

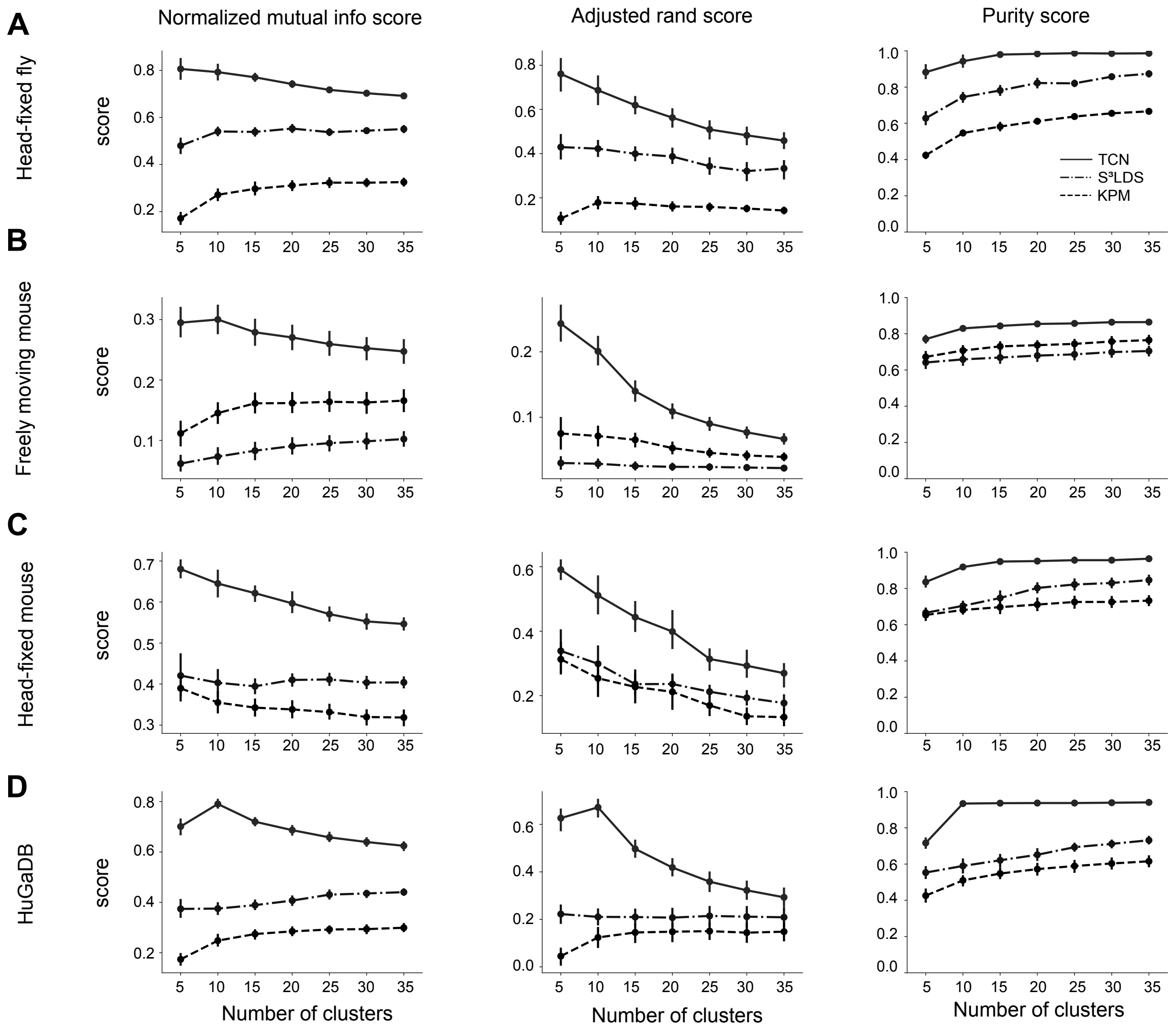


Figure S6: **The latent spaces of models are shaped by the extent to which labels are used in training: position-velocity features.** Cluster metrics for models trained with position-velocity features. These cluster metrics – including normalized mutual info score, adjusted rand score, and purity score – complement the cluster homogeneity score in Fig. 5. The addition of hand labels produces more homogeneous clusters in the models' latent spaces.

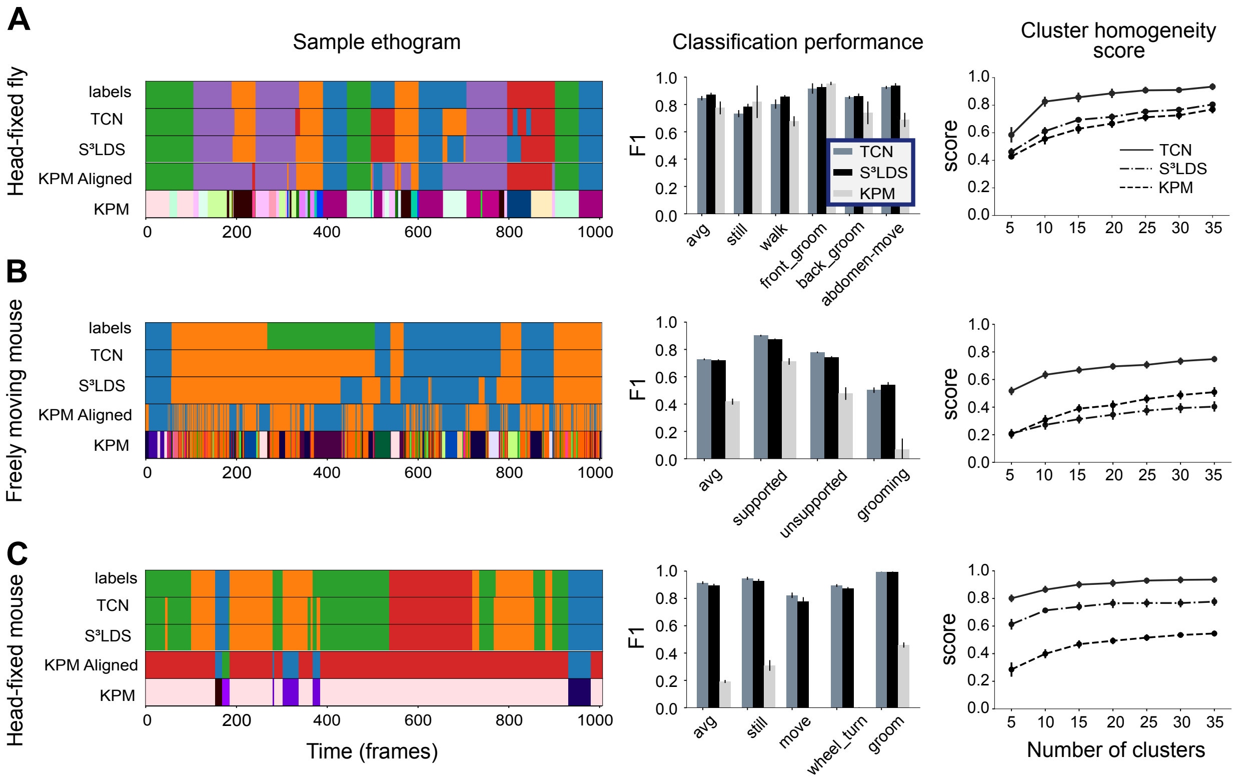


Figure S7: **Keypoint-MoSeq performance on non-fly datasets: position features.** Comparisons between the models using only position features. Conventions as in Fig. 6. We find the TCN, which is purely supervised, achieves the highest alignment of the latent space with the ground truth labels as measured by the cluster homogeneity score.

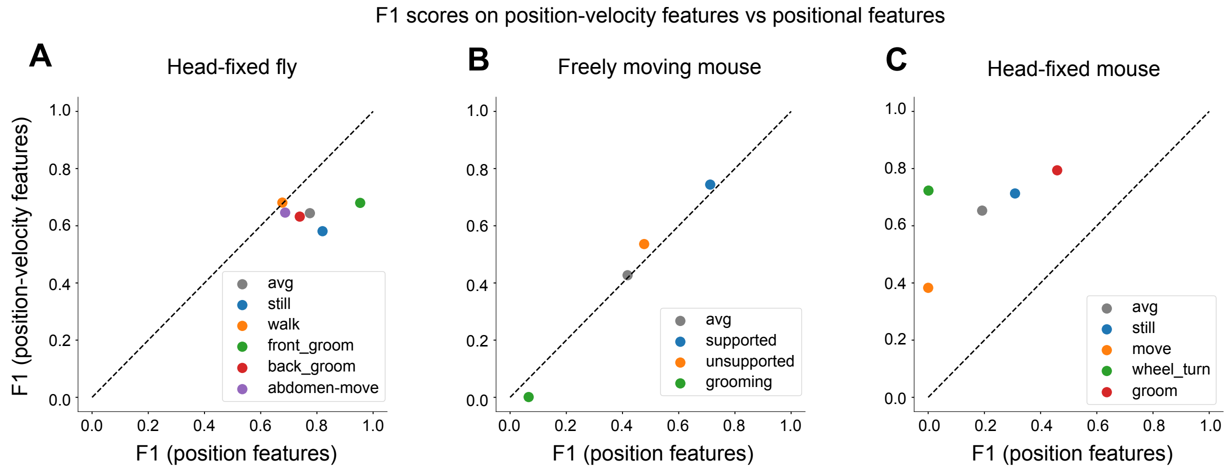


Figure S8: **Keypoint-MoSeq performance: position-velocity features vs position features.** Comparisons between the keypoint-Moseq models trained on position-velocity features vs position features. We find that keypoint-Moseq performs best with the position features on the head-fixed fly dataset, and the position-velocity features on the two mouse datasets.

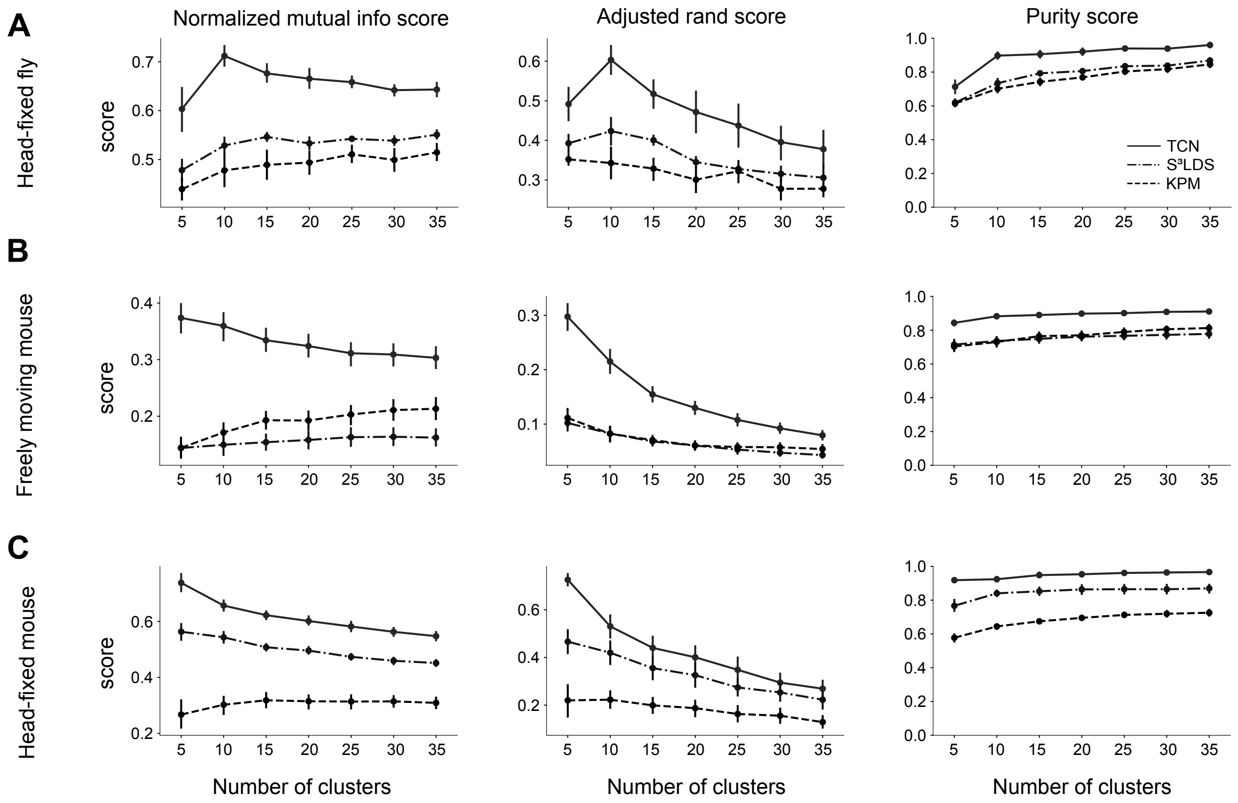


Figure S9: **Cluster metrics for model latent spaces: position features.** Cluster metrics for models trained with position features. Conventions as in Fig. S6.

## 6 Supplementary tables

| | Experiment ID | Total frames | Walk | Still | Front groom | Back groom | Abdomen move |
|---|---|---|---|---|---|---|---|
| **Train** | 2019_08_07_fly2 | 50000 | 300 | 300 | 100 | 300 | 591 |
| | 2019_08_08_fly1 | 94960 | 300 | 300 | 300 | 150 | 594 |
| | 2019_08_20_fly2 | 65108 | 300 | 300 | 300 | 300 | 1067 |
| | 2019_10_10_fly3 | 141042 | 300 | 300 | 300 | 300 | 478 |
| | 2019_10_14_fly3 | 140929 | 300 | 300 | 300 | 300 | 318 |
| **Test** | 2019_06_26_fly2 | 45000 | 300 | 300 | 300 | 300 | 706 |
| | 2019_08_14_fly1 | 124144 | 300 | 300 | 300 | 300 | 693 |
| | 2019_08_20_fly3 | 73055 | 300 | 300 | 300 | 300 | 405 |
| | 2019_10_14_fly2 | 139925 | 300 | 300 | 300 | 300 | 101 |
| | 2019_10_21_fly1 | 142554 | 300 | 300 | 300 | 300 | 0 |
| | **Totals** | 1016717 | 3000 | 3000 | 2800 | 2850 | 4953 |

Table S1: The number of labeled frames per behavior for the head-fixed fly dataset.

| | Experiment ID | Total frames | Supported rear | Unsupported rear | Groom |
|---|---|---|---|---|---|
| **Train** | OFT_5 | 14000 | 3267 | 1208 | 387 |
| | OFT_6 | 14000 | 2424 | 1090 | 428 |
| | OFT_11 | 14000 | 1900 | 1036 | 117 |
| | OFT_12 | 14000 | 2354 | 2213 | 202 |
| | OFT_14 | 14000 | 3165 | 2071 | 140 |
| | OFT_15 | 14000 | 2038 | 1433 | 154 |
| | OFT_16 | 14000 | 1773 | 826 | 493 |
| | OFT_23 | 14000 | 1708 | 263 | 396 |
| | OFT_24 | 14000 | 2130 | 2312 | 298 |
| | OFT_38 | 14000 | 2564 | 2598 | 513 |
| **Test** | OFT_39 | 14000 | 2159 | 2062 | 570 |
| | OFT_41 | 14000 | 2381 | 1479 | 178 |
| | OFT_43 | 14000 | 3642 | 338 | 125 |
| | OFT_44 | 14000 | 2314 | 1069 | 139 |
| | OFT_49 | 14000 | 2638 | 1120 | 142 |
| | OFT_50 | 14000 | 1550 | 873 | 386 |
| | OFT_51 | 14000 | 1799 | 782 | 442 |
| | OFT_52 | 14000 | 2949 | 1605 | 547 |
| | OFT_54 | 14000 | 2587 | 2012 | 367 |
| | OFT_58 | 14000 | 2468 | 1858 | 577 |
| | **Totals** | 280000 | 47810 | 28248 | 6601 |

Table S2: The number of labeled frames per behavior for the freely moving mouse dataset.

| | Experiment ID | Total frames | Still | Move | Wheel turn | Groom |
|---|---|---|---|---|---|---|
| **Train** | danlab_DY_009_2020-02-27-001 | 100000 | 497 | 316 | 374 | 300 |
| | danlab_DY_018_2020-10-15-001 | 100000 | 400 | 268 | 241 | 580 |
| | hoferlab_SWC_061_2020-11-23-001 | 100000 | 320 | 261 | 494 | 265 |
| | mrsicflogellab_SWC_058_2020-12-11-001 | 100000 | 433 | 357 | 358 | 298 |
| | wittenlab_ibl_witten_26_2021-01-27-002 | 100000 | 624 | 235 | 353 | 360 |
| **Test** | churchlandlab_CSHL045_2020-02-27-001 | 100000 | 306 | 368 | 522 | 469 |
| | cortexlab_KS020_2020-02-06-001 | 100000 | 378 | 340 | 322 | 208 |
| | hoferlab_SWC_043_2020-09-15-001 | 100000 | 469 | 363 | 306 | 513 |
| | mrsicflogellab_SWC_052_2020-10-22-001 | 100000 | 338 | 179 | 494 | 242 |
| | wittenlab_ibl_witten_27_2021-01-21-001 | 100000 | 335 | 35 | 536 | 50 |
| | **Totals** | 1000000 | 4100 | 2722 | 4000 | 3285 |

Table S3: The number of labeled frames per behavior for the IBL head-fixed mouse dataset.

| | Experiment ID | Total frames | Walking | Running | Going up | Going down | Sitting | Sitting down | Standing up | Standing |
|---|---|---|---|---|---|---|---|---|---|---|
| **Train** | subject 05 | 56199 | 23034 | 2154 | 9820 | 10246 | 4215 | 834 | 1052 | 4844 |
| | subject 07 | 53571 | 23533 | 4111 | 5767 | 6460 | 5000 | 1164 | 1273 | 6263 |
| | subject 09 | 40066 | 12254 | 3712 | 7374 | 5954 | 3278 | 1038 | 1227 | 5229 |
| | subject 12 | 52005 | 14343 | 5796 | 7683 | 7805 | 6843 | 908 | 1085 | 7542 |
| | subject 14 | 55366 | 23365 | 5529 | 8829 | 6199 | 4771 | 920 | 900 | 4853 |
| **Test** | subject 06 | 67747 | 33386 | 11334 | 5367 | 6214 | 3153 | 922 | 1008 | 6363 |
| | subject 08 | 49093 | 17327 | 4081 | 7138 | 6262 | 4710 | 1101 | 1509 | 6965 |
| | subject 11 | 45137 | 16202 | 3727 | 7768 | 5797 | 4471 | 1257 | 1329 | 4586 |
| | subject 13 | 52053 | 17971 | 5374 | 10969 | 6942 | 3896 | 1095 | 1314 | 4492 |
| | subject 17 | 45547 | 12579 | 1862 | 8723 | 6130 | 6867 | 1276 | 1235 | 6875 |
| | **Totals** | 516784 | 193994 | 47680 | 79438 | 68009 | 47204 | 10515 | 11932 | 58012 |

Table S4: The number of labeled frames per behavior for the human gait database.

# Appendix A Variational lower bound of S³LDS model

## A.1 All labels observed

We first derive the variational lower bound, assuming all $y_t$ are observed. For clarity we drop the dependence on $\theta$ and $\phi$.

$$\begin{aligned}\log p(\mathbf{x}_{1:T}, y_{1:T}) &\geq \int_{\mathbf{z}_{1:T}} q(\mathbf{z}_{1:T}|\mathbf{x}_{1:T}, y_{1:T}) \log \frac{p(\mathbf{x}_{1:T}|\mathbf{z}_{1:T}) p(\mathbf{z}_{1:T}, y_{1:T})}{q(\mathbf{z}_{1:T}|\mathbf{x}_{1:T}, y_{1:T})} \\ &= \mathbb{E}_{q(\mathbf{z}_{1:T}|\mathbf{x}_{1:T}, y_{1:T})}\left[\log p(\mathbf{x}_{1:T}|\mathbf{z}_{1:T})\right] + \mathbb{E}_{q(\mathbf{z}_{1:T}|\mathbf{x}_{1:T}, y_{1:T})}\left[\log \frac{p(\mathbf{z}_{1:T}, y_{1:T})}{q(\mathbf{z}_{1:T}|\mathbf{x}_{1:T}, y_{1:T})}\right].\end{aligned}$$

For the first term, recall that both $q(\mathbf{z}_{1:T}|\mathbf{x}_{1:T}, y_{1:T})$ and $p(\mathbf{x}_{1:T}|\mathbf{z}_{1:T})$ factorize across time:

$$\begin{aligned}\mathbb{E}_{q(\mathbf{z}_{1:T}|\mathbf{x}_{1:T}, y_{1:T})}\left[\log p(\mathbf{x}_{1:T}|\mathbf{z}_{1:T})\right] &= \int_{\mathbf{z}_{1:T}} \prod_{t=1}^{T} q(\mathbf{z}_t|\mathbf{x}_{\mathcal{T}_t}, y_t) \log \prod_{t=1}^{T} p(\mathbf{x}_t|\mathbf{z}_t) \\ &= \sum_{t=1}^{T} \int_{\mathbf{z}_{1:T}} \prod_{t=1}^{T} q(\mathbf{z}_t|\mathbf{x}_{\mathcal{T}_t}, y_t) \log p(\mathbf{x}_t|\mathbf{z}_t) \\ &= \sum_{t=1}^{T} \mathbb{E}_{q(\mathbf{z}_t|\mathbf{x}_{\mathcal{T}_t}, y_t)}\left[\log p(\mathbf{x}_t|\mathbf{z}_t)\right]. \end{aligned} \tag{27}$$

For the second term,

$$\begin{aligned}&\mathbb{E}_{q(\mathbf{z}_{1:T}|\mathbf{x}_{1:T}, y_{1:T})}\left[\log \frac{p(\mathbf{z}_{1:T}, y_{1:T})}{q(\mathbf{z}_{1:T}|\mathbf{x}_{1:T}, y_{1:T})}\right] = \\ &\int_{\mathbf{z}_{1:T}} \prod_{t=1}^{T} q(\mathbf{z}_t|\mathbf{x}_{\mathcal{T}_t}, y_t) \log \frac{p(\mathbf{z}_1)p(y_1)\prod_{t=2}^{T} p(\mathbf{z}_t|\mathbf{z}_{t-1}, y_t) p(y_t|y_{t-1}, \mathbf{z}_{t-1})}{\prod_{t=1}^{T} q(\mathbf{z}_t|\mathbf{x}_{\mathcal{T}_t}, y_t)} \\ &= \int_{\mathbf{z}_{1:T}} \prod_{t=1}^{T} q(\mathbf{z}_t|\mathbf{x}_{\mathcal{T}_t}, y_t) \log \frac{p(\mathbf{z}_1)p(y_1)}{q(\mathbf{z}_1|\mathbf{x}_{\mathcal{T}_1}, y_1)} \\ &\quad + \sum_{t=2}^{T} \int_{\mathbf{z}_{1:T}} \prod_{t=1}^{T} q(\mathbf{z}_t|\mathbf{x}_{\mathcal{T}_t}, y_t) \log \frac{p(\mathbf{z}_t|\mathbf{z}_{t-1}, y_t) p(y_t|y_{t-1}, \mathbf{z}_{t-1})}{q(\mathbf{z}_t|\mathbf{x}_{\mathcal{T}_t}, y_t)} \\ &= \int_{\mathbf{z}_1} q(\mathbf{z}_1|\mathbf{x}_{\mathcal{T}_1}, y_1) \log \frac{p(\mathbf{z}_1)p(y_1)}{q(\mathbf{z}_1|\mathbf{x}_{\mathcal{T}_1}, y_1)} \\ &\quad + \sum_{t=2}^{T} \int_{\mathbf{z}_{t-1}} \int_{\mathbf{z}_t} q(\mathbf{z}_{t-1}|\mathbf{x}_{\mathcal{T}_{t-1}}, y_{t-1}) q(\mathbf{z}_t|\mathbf{x}_{\mathcal{T}_t}, y_t) \log \frac{p(\mathbf{z}_t|\mathbf{z}_{t-1}, y_t) p(y_t|y_{t-1}, \mathbf{z}_{t-1})}{q(\mathbf{z}_t|\mathbf{x}_{\mathcal{T}_t}, y_t)} \\ &= \log p(y_1) - \mathrm{KL}\left[q(\mathbf{z}_1|\mathbf{x}_{\mathcal{T}_1}, y_1) || p(\mathbf{z}_1)\right] - \sum_{t=2}^{T} \mathbb{E}_{q(\mathbf{z}_{t-1}|\mathbf{x}_{\mathcal{T}_{t-1}}, y_{t-1})}\left[\mathrm{KL}\left[q(\mathbf{z}_t|\mathbf{x}_{\mathcal{T}_t}, y_t) || p(\mathbf{z}_t|\mathbf{z}_{t-1}, y_t)\right]\right] \\ &\quad + \sum_{t=2}^{T} \mathbb{E}_{q(\mathbf{z}_{t-1}|\mathbf{x}_{\mathcal{T}_{t-1}}, y_{t-1})}\left[\log p(y_t|y_{t-1}, \mathbf{z}_{t-1})\right]. \end{aligned} \tag{28}$$

Now we combine Eqs. 27 and 28 to get the full variational lower bound:

$$\begin{aligned}\log p(\mathbf{x}_{1:T}, y_{1:T}) \geq &\sum_{t=1}^{T} \mathbb{E}_{q(\mathbf{z}_t|\mathbf{x}_{\mathcal{T}_t}, y_t)} \left[\log p(\mathbf{x}_t|\mathbf{z}_t)\right] \\ &- \mathrm{KL}\left[q(\mathbf{z}_1|\mathbf{x}_{\mathcal{T}_1}, y_1)||p(\mathbf{z}_1)\right] + \log p(y_1) \\ &- \sum_{t=2}^{T} \mathbb{E}_{q(\mathbf{z}_{t-1}|\mathbf{x}_{\mathcal{T}_{t-1}}, y_{t-1})} \left[\mathrm{KL}\left[q(\mathbf{z}_t|\mathbf{x}_{\mathcal{T}_t}, y_t)||p(\mathbf{z}_t|\mathbf{z}_{t-1}, y_t)\right]\right] \\ &+ \sum_{t=2}^{T} \mathbb{E}_{q(\mathbf{z}_{t-1}|\mathbf{x}_{\mathcal{T}_{t-1}}, y_{t-1})} \left[\log p(y_t|y_{t-1}, \mathbf{z}_{t-1})\right] \qquad (29) \\ \approx &\sum_{t=1}^{T} \log p(\mathbf{x}_t|\tilde{\mathbf{z}}_t) \\ &- \mathrm{KL}\left[q(\mathbf{z}_1|\mathbf{x}_{\mathcal{T}_1}, y_1)||p(\mathbf{z}_1)\right] + \log p(y_1) \\ &- \sum_{t=2}^{T} \mathrm{KL}\left[q(\mathbf{z}_t|\mathbf{x}_{\mathcal{T}_t}, y_t)||p(\mathbf{z}_t|\tilde{\mathbf{z}}_{t-1}, y_t)\right] \\ &+ \sum_{t=2}^{T} \log p(y_t|y_{t-1}, \tilde{\mathbf{z}}_{t-1}). \qquad (30)\end{aligned}$$

### A.2 All labels unobserved

Next, we derive the variational lower bound for the more complicated case where we assume all $y_t$ are *un*observed.

$$\begin{aligned}\log p(\mathbf{x}_{1:T}) \geq &\int_{\mathbf{z}_{1:T}} \int_{y_{1:T}} q(\mathbf{z}_{1:T}, y_{1:T}|\mathbf{x}_{1:T}) \log \frac{p(\mathbf{x}_{1:T}|\mathbf{z}_{1:T})p(\mathbf{z}_{1:T}, y_{1:T})}{q(\mathbf{z}_{1:T}, y_{1:T}|\mathbf{x}_{1:T})} \\ = &\int_{\mathbf{z}_{1:T}} \int_{y_{1:T}} q(\mathbf{z}_{1:T}, y_{1:T}|\mathbf{x}_{1:T}) \log p(\mathbf{x}_{1:T}|\mathbf{z}_{1:T}) \\ &+ \int_{\mathbf{z}_{1:T}} \int_{y_{1:T}} q(\mathbf{z}_{1:T}, y_{1:T}|\mathbf{x}_{1:T}) \log \frac{p(\mathbf{z}_{1:T}, y_{1:T})}{q(\mathbf{z}_{1:T}, y_{1:T}|\mathbf{x}_{1:T})}\end{aligned}$$

Again, recall that both $q(\mathbf{z}_{1:T}|\mathbf{x}_{1:T}, y_{1:T})$ and $p(\mathbf{x}_{1:T}|\mathbf{z}_{1:T})$ factorize across time. We now expand the first term:

$$\begin{aligned}&\int_{\mathbf{z}_{1:T}} \int_{y_{1:T}} q(\mathbf{z}_{1:T}, y_{1:T}|\mathbf{x}_{1:T}) \log p(\mathbf{x}_{1:T}|\mathbf{z}_{1:T}) = \\ &\int_{\mathbf{z}_{1:T}} \int_{y_{1:T}} \prod_{t=1}^{T} q(y_t|\mathbf{x}_{\mathcal{T}_t}) q(\mathbf{z}_t|\mathbf{x}_{\mathcal{T}_t}, y_t) \log \prod_{t=1}^{T} p(\mathbf{x}_t|\mathbf{z}_t) \\ = &\sum_{t=1}^{T} \int_{\mathbf{z}_{1:T}} \int_{y_{1:T}} \prod_{t=1}^{T} q(y_t|\mathbf{x}_{\mathcal{T}_t}) q(\mathbf{z}_t|\mathbf{x}_{\mathcal{T}_t}, y_t) \log p(\mathbf{x}_t|\mathbf{z}_t) \\ = &\sum_{t=1}^{T} \int_{\mathbf{z}_t} \int_{y_t} q(y_t|\mathbf{x}_{\mathcal{T}_t}) q(\mathbf{z}_t|\mathbf{x}_{\mathcal{T}_t}, y_t) \log p(\mathbf{x}_t|\mathbf{z}_t) \\ = &\sum_{t=1}^{T} \mathbb{E}_{q(y_t|\mathbf{x}_{\mathcal{T}_t}) q(\mathbf{z}_t|\mathbf{x}_{\mathcal{T}_t}, y_t)} \left[\log p(\mathbf{x}_t|\mathbf{z}_t)\right]. \qquad (31)\end{aligned}$$

For the second term,

$$
\begin{aligned}
&\int_{\mathbf{z}_{1:T}}\int_{y_{1:T}} q(\mathbf{z}_{1:T}, y_{1:T}|\mathbf{x}_{1:T}) \log \frac{p(\mathbf{z}_{1:T}, y_{1:T})}{q(\mathbf{z}_{1:T}, y_{1:T}|\mathbf{x}_{1:T})} \\
=&\int_{\mathbf{z}_{1:T}}\int_{y_{1:T}} \prod_{t=1}^{T} q(y_t|\mathbf{x}_{\mathcal{T}_t})q(\mathbf{z}_t|\mathbf{x}_{\mathcal{T}_t}, y_t) \log \frac{p(\mathbf{z}_1)p(y_1)\prod_{t=2}^{T} p(\mathbf{z}_t|\mathbf{z}_{t-1}, y_t)p(y_t|y_{t-1}, \mathbf{z}_{t-1})}{\prod_{t=1}^{T} q(y_t|\mathbf{x}_{\mathcal{T}_t})q(\mathbf{z}_t|\mathbf{x}_{\mathcal{T}_t}, y_t)} \\
=&\int_{\mathbf{z}_{1:T}}\int_{y_{1:T}} \prod_{t=1}^{T} q(y_t|\mathbf{x}_{\mathcal{T}_t})q(\mathbf{z}_t|\mathbf{x}_{\mathcal{T}_t}, y_t) \log \frac{p(\mathbf{z}_1)p(y_1)}{q(\mathbf{z}_1|\mathbf{x}_{\mathcal{T}_1}, y_1)q(y_1|\mathbf{x}_{\mathcal{T}_1})} \\
&+\int_{\mathbf{z}_{1:T}}\int_{y_{1:T}} \prod_{t=1}^{T} q(y_t|\mathbf{x}_{\mathcal{T}_t})q(\mathbf{z}_t|\mathbf{x}_{\mathcal{T}_t}, y_t) \left[\sum_{t=2}^{T} \log \frac{p(\mathbf{z}_t|\mathbf{z}_{t-1}, y_t)p(y_t|y_{t-1}, \mathbf{z}_{t-1})}{q(y_t|\mathbf{x}_{\mathcal{T}_t})q(\mathbf{z}_t|\mathbf{x}_{\mathcal{T}_t}, y_t)}\right] \\
=&\int_{\mathbf{z}_1}\int_{y_1} q(\mathbf{z}_1|\mathbf{x}_{\mathcal{T}_1}, y_1)q(y_1|\mathbf{x}_{\mathcal{T}_1}) \log \frac{p(\mathbf{z}_1)p(y_1)}{q(\mathbf{z}_1|\mathbf{x}_{\mathcal{T}_1}, y_1)q(y_1|\mathbf{x}_{\mathcal{T}_1})} \\
&+\sum_{t=2}^{T}\int_{\mathbf{z}_{t-1}}\int_{\mathbf{z}_t}\int_{y_{t-1}}\int_{y_t} q(y_t|\mathbf{x}_{\mathcal{T}_t})q(y_{t-1}|\mathbf{x}_{\mathcal{T}_{t-1}})q(\mathbf{z}_t|\mathbf{x}_{\mathcal{T}_t}, y_t)q(\mathbf{z}_{t-1}|\mathbf{x}_{\mathcal{T}_{t-1}}, y_{t-1}) \log \frac{p(\mathbf{z}_t|\mathbf{z}_{t-1}, y_t)}{q(\mathbf{z}_t|\mathbf{x}_{\mathcal{T}_t}, y_t)} \\
&+\sum_{t=2}^{T}\int_{\mathbf{z}_{t-1}}\int_{\mathbf{z}_t}\int_{y_{t-1}}\int_{y_t} q(y_t|\mathbf{x}_{\mathcal{T}_t})q(y_{t-1}|\mathbf{x}_{\mathcal{T}_{t-1}})q(\mathbf{z}_t|\mathbf{x}_{\mathcal{T}_t}, y_t)q(\mathbf{z}_{t-1}|\mathbf{x}_{\mathcal{T}_{t-1}}, y_{t-1}) \log \frac{p(y_t|y_{t-1}, \mathbf{z}_{t-1})}{q(y_t|\mathbf{x}_{\mathcal{T}_t})}. \qquad (32)
\end{aligned}
$$

The first term from Eq. 32 becomes

$$
\begin{aligned}
&\int_{\mathbf{z}_1}\int_{y_1} q(\mathbf{z}_1|\mathbf{x}_{\mathcal{T}_1}, y_1)q(y_1|\mathbf{x}_{\mathcal{T}_1}) \log \frac{p(\mathbf{z}_1)p(y_1)}{q(\mathbf{z}_1|\mathbf{x}_{\mathcal{T}_1}, y_1)q(y_1|\mathbf{x}_{\mathcal{T}_1})} \\
=&\int_{y_1} q(y_1|\mathbf{x}_{\mathcal{T}_1})\int_{\mathbf{z}_1} q(\mathbf{z}_1|\mathbf{x}_{\mathcal{T}_1}, y_1) \log \frac{p(\mathbf{z}_1)}{q(\mathbf{z}_1|\mathbf{x}_{\mathcal{T}_1}, y_1)} + \int_{y_1} q(y_1|\mathbf{x}_{\mathcal{T}_1}) \log \frac{p(y_1)}{q(y_1|\mathbf{x}_{\mathcal{T}_1})}\int_{\mathbf{z}_1} q(\mathbf{z}_1|\mathbf{x}_{\mathcal{T}_1}, y_1) \\
=&-\mathbb{E}_{q(y_1|\mathbf{x}_{\mathcal{T}_1})}\left[\mathrm{KL}\left[q(\mathbf{z}_1|\mathbf{x}_{\mathcal{T}_1}, y_1)||p(\mathbf{z}_1)\right]\right] - \mathrm{KL}\left[q(y_1|\mathbf{x}_{\mathcal{T}_1})||p(y_1)\right]. \qquad (33)
\end{aligned}
$$

The second term from Eq. 32 becomes

$$
\begin{aligned}
&\sum_{t=2}^{T}\int_{\mathbf{z}_{t-1}}\int_{\mathbf{z}_t}\int_{y_{t-1}}\int_{y_t} q(y_t|\mathbf{x}_{\mathcal{T}_t})q(y_{t-1}|\mathbf{x}_{\mathcal{T}_{t-1}})q(\mathbf{z}_t|\mathbf{x}_{\mathcal{T}_t}, y_t)q(\mathbf{z}_{t-1}|\mathbf{x}_{\mathcal{T}_{t-1}}, y_{t-1}) \log \frac{p(\mathbf{z}_t|\mathbf{z}_{t-1}, y_t)}{q(\mathbf{z}_t|\mathbf{x}_{\mathcal{T}_t}, y_t)} \\
=&-\sum_{t=2}^{T}\mathbb{E}_{q(y_t|\mathbf{x}_{\mathcal{T}_t})q(y_{t-1}|\mathbf{x}_{\mathcal{T}_{t-1}})q(\mathbf{z}_{t-1}|\mathbf{x}_{\mathcal{T}_{t-1}}, y_{t-1})}\left[\mathrm{KL}\left[q(\mathbf{z}_t|\mathbf{x}_{\mathcal{T}_t}, y_t)||p(\mathbf{z}_t|\mathbf{z}_{t-1}, y_t)\right]\right] \qquad (34)
\end{aligned}
$$

and the third term from Eq. 32 becomes

$$
\begin{aligned}
&\sum_{t=2}^{T}\int_{\mathbf{z}_{t-1}}\int_{y_{t-1}}\int_{y_t} q(y_t|\mathbf{x}_{\mathcal{T}_t})q(y_{t-1}|\mathbf{x}_{\mathcal{T}_{t-1}})q(\mathbf{z}_{t-1}|\mathbf{x}_{\mathcal{T}_{t-1}}, y_{t-1}) \log \frac{p(y_t|y_{t-1}, \mathbf{z}_{t-1})}{q(y_t|\mathbf{x}_{\mathcal{T}_t})} \\
=&-\sum_{t=2}^{T}\mathbb{E}_{q(y_{t-1}|\mathbf{x}_{\mathcal{T}_{t-1}})q(\mathbf{z}_{t-1}|\mathbf{x}_{\mathcal{T}_{t-1}}, y_{t-1})}\left[\mathrm{KL}\left[q(y_t|\mathbf{x}_{\mathcal{T}_t})||p(y_t|y_{t-1}, \mathbf{z}_{t-1})\right]\right]. \qquad (35)
\end{aligned}
$$

Now we combine Eqs. 31, 33, 34, and 35 to get the full variational lower bound:

$$
\begin{aligned}
\log p(\mathbf{x}_{1:T}) \geq& \sum_{t=1}^{T} \mathbb{E}_{q(y_t|\mathbf{x}_{\mathcal{T}_t})q(\mathbf{z}_t|\mathbf{x}_{\mathcal{T}_t},y_t)} \left[\log p(\mathbf{x}_t|\mathbf{z}_t)\right] \\
&- \mathbb{E}_{q(y_1|\mathbf{x}_{\mathcal{T}_1})} \left[\mathrm{KL}\left[q(\mathbf{z}_1|\mathbf{x}_{\mathcal{T}_1}, y_1)||p(\mathbf{z}_1)\right]\right] - \mathrm{KL}\left[q(y_1|\mathbf{x}_{\mathcal{T}_1})||p(y_1)\right] \\
&- \sum_{t=2}^{T} \mathbb{E}_{q(y_t|\mathbf{x}_{\mathcal{T}_t})q(y_{t-1}|\mathbf{x}_{\mathcal{T}_{t-1}})q(\mathbf{z}_{t-1}|\mathbf{x}_{\mathcal{T}_{t-1}},y_{t-1})} \left[\mathrm{KL}\left[q(\mathbf{z}_t|\mathbf{x}_{\mathcal{T}_t}, y_t)||p(\mathbf{z}_t|\mathbf{z}_{t-1}, y_t)\right]\right] \\
&- \sum_{t=2}^{T} \mathbb{E}_{q(y_{t-1}|\mathbf{x}_{\mathcal{T}_{t-1}})q(\mathbf{z}_{t-1}|\mathbf{x}_{\mathcal{T}_{t-1}},y_{t-1})} \left[\mathrm{KL}\left[q(y_t|\mathbf{x}_{\mathcal{T}_t})||p(y_t|y_{t-1}, \mathbf{z}_{t-1})\right]\right] \\
=& \sum_{t=1}^{T}\sum_{k=1}^{K} \Big(q(y_t = k|\mathbf{x}_{\mathcal{T}_t})\mathbb{E}_{q(\mathbf{z}_t|\mathbf{x}_{\mathcal{T}_t},y_t=k)}[\log p(\mathbf{x}_t|\mathbf{z}_t)]\Big) \\
&- \sum_{k=1}^{K} \Big(q(y_1 = k|\mathbf{x}_{\mathcal{T}_1})\mathrm{KL}\left[q(\mathbf{z}_1|\mathbf{x}_{\mathcal{T}_1}, y_1 = k)||p(\mathbf{z}_1)\right]\Big) - \mathrm{KL}\left[q(y_1|\mathbf{x}_{\mathcal{T}_1})||p(y_1)\right] \\
&- \sum_{t=2}^{T}\sum_{k=1}^{K}\sum_{k'=1}^{K} \Big(q(y_t = k|\mathbf{x}_{\mathcal{T}_t})q(y_{t-1} = k'|\mathbf{x}_{\mathcal{T}_{t-1}}) \\
&\qquad \mathbb{E}_{q(\mathbf{z}_{t-1}|\mathbf{x}_{\mathcal{T}_{t-1}},y_{t-1}=k')}[\mathrm{KL}\left[q(\mathbf{z}_t|\mathbf{x}_{\mathcal{T}_t}, y_t = k)||p(\mathbf{z}_t|\mathbf{z}_{t-1}, y_t = k)\right]]\Big) \\
&- \sum_{t=2}^{T}\sum_{k=1}^{K} \Big(q(y_{t-1} = k|\mathbf{x}_{\mathcal{T}_{t-1}})\mathbb{E}_{q(\mathbf{z}_{t-1}|\mathbf{x}_{\mathcal{T}_{t-1}},y_{t-1}=k)}[\mathrm{KL}\left[q(y_t|\mathbf{x}_{\mathcal{T}_t})||p(y_t|y_{t-1} = k, \mathbf{z}_{t-1})\right]]\Big) \qquad (36) \\
\approx& \sum_{t=1}^{T}\sum_{k=1}^{K} \Big(q(y_t = k|\mathbf{x}_{\mathcal{T}_t})\log p(\mathbf{x}_t|\tilde{\mathbf{z}}_t^k)\Big) \\
&- \sum_{k=1}^{K} \Big(q(y_1 = k|\mathbf{x}_{\mathcal{T}_t})\mathrm{KL}\left[q(\mathbf{z}_1|\mathbf{x}_{\mathcal{T}_1}, y_1 = k)||p(\mathbf{z}_1)\right]\Big) - \mathrm{KL}\left[q(y_1|\mathbf{x}_{\mathcal{T}_1})||p(y_1)\right] \\
&- \sum_{t=2}^{T}\sum_{k=1}^{K}\sum_{k'=1}^{K} \Big(q(y_t = k|\mathbf{x}_{\mathcal{T}_t})q(y_{t-1} = k'|\mathbf{x}_{\mathcal{T}_{t-1}}) \\
&\qquad \mathrm{KL}\Big[q(\mathbf{z}_t|\mathbf{x}_{\mathcal{T}_t}, y_t = k)||p(\mathbf{z}_t|\tilde{\mathbf{z}}_{t-1}^{k'}, y_t = k)\Big]\Big) \\
&- \sum_{t=2}^{T}\sum_{k=1}^{K} \Big(q(y_{t-1} = k|\mathbf{x}_{\mathcal{T}_{t-1}})\mathrm{KL}\left[q(y_t|\mathbf{x}_{\mathcal{T}_t})||p(y_t|y_{t-1} = k, \tilde{\mathbf{z}}_{t-1}^k)\right]\Big) \qquad (37)
\end{aligned}
$$

where

$$
\tilde{\mathbf{z}}_t^k \sim q(\mathbf{z}_t|\mathbf{x}_{\mathcal{T}_t}, y_t = k) \text{ and } \tilde{\mathbf{z}}_{t-1}^k \sim q(\mathbf{z}_{t-1}|\mathbf{x}_{\mathcal{T}_{t-1}}, y_{t-1} = k).
$$

### A.3 Mix of observed and unobserved labels

The loss function that incorporates both observed and unobserved discrete labels can be computed in an elegant and straightforward manner. For notational simplicity we define a new distribution over the discrete variables, which is equal to the approximate posterior for unlabeled time points, and equal to a one-hot vector representing the true discrete state for labeled time points. Let $\mathcal{T}_\mathcal{L}$ be the set of all labeled time points and $\mathcal{T}_\mathcal{U}$ be the set of all unlabeled time points, such that $\mathcal{T}_\mathcal{L} \cup \mathcal{T}_\mathcal{U} = \{1, \ldots, T\}$ and $\mathcal{T}_\mathcal{L} \cap \mathcal{T}_\mathcal{U} = \varnothing$. Then we define

$$
r(y_t|\mathbf{x}_{\mathcal{T}_t}) = \begin{cases} e_{y_t} & \text{if } t \in \mathcal{T}_\mathcal{L} \\ q(y_t|\mathbf{x}_{\mathcal{T}_t}) & \text{if } t \in \mathcal{T}_\mathcal{U} \end{cases}
$$

where $e_{y_t}$ denotes a vector with a 1 in the $y_t^{\text{th}}$ coordinate and 0 elsewhere. We can then compute Eq. 37 using this mixture of distributions due to the fact that each of the individual terms in the unsupervised ELBO (Eq. 16) reduces to the corresponding term in the supervised ELBO (Eq. 15) when using one-hot vectors. In addition, we compute the classification loss on the approximate posterior $q(y_t|\mathbf{x}_{\mathcal{T}_t})$ for all observed discrete labels:

$$\begin{aligned}
\mathcal{L}_{ss} = & \sum_{t=1}^{T}\sum_{k=1}^{K}\Big(r(y_t = k|\mathbf{x}_{\mathcal{T}_t})\log p(\mathbf{x}_t|\tilde{\mathbf{z}}_t^k)\Big) \\
& - \sum_{k=1}^{K}\Big(r(y_1 = k|\mathbf{x}_{\mathcal{T}_1})\mathrm{KL}\left[q(\mathbf{z}_1|\mathbf{x}_{\mathcal{T}_1}, y_1 = k)||p(\mathbf{z}_1)\right]\Big) - \mathrm{KL}\left[r(y_1|\mathbf{x}_{\mathcal{T}_1})||p(y_1)\right] \\
& - \sum_{t=2}^{T}\sum_{k=1}^{K}\sum_{k'=1}^{K}\Big(r(y_t = k|\mathbf{x}_{\mathcal{T}_t})r(y_{t-1} = k'|\mathbf{x}_{\mathcal{T}_{t-1}}) \\
& \qquad \mathrm{KL}\Big[q(\mathbf{z}_t|\mathbf{x}_{\mathcal{T}_t}, y_t = k)||p(\mathbf{z}_t|\tilde{\mathbf{z}}_{t-1}^{k'}, y_t = k)\Big]\Big) \\
& - \sum_{t=2}^{T}\sum_{k=1}^{K}\Big(r(y_{t-1} = k|\mathbf{x}_{\mathcal{T}_{t-1}})\mathrm{KL}\left[r(y_t|\mathbf{x}_{\mathcal{T}_t})||p(y_t|y_{t-1} = k, \tilde{\mathbf{z}}_{t-1}^k)\right]\Big) \\
& + \sum_{t\in\mathcal{T}_{\mathcal{L}}}\alpha\log q(y_t|\mathbf{x}_{\mathcal{T}_t}).
\end{aligned} \tag{38}$$

## Appendix B The International Brain Laboratory consortium members

Larry Abbot[1], Luigi Acerbi[2], Valeria Aguillon-Rodriguez[3], Mandana Ahmadi[4], Jaweria Amjad[4], Dora Angelaki[5], Jaime Arlandis[6], Zoe C Ashwood[7], Kush Banga[8], Hailey Barrell[9], Hannah M Bayer[1], Brandon Benson[10], Julius Benson[5], Jai Bhagat[8], Dan Birman[9], Niccolò Bonacchi[6], Kcenia Bougrova[6], Julien Boussard[1], Sebastian A Bruijns[11], E Kelly Buchanan[1], Robert Campbell[12], Matteo Carandini[13], Joana A Catarino[6], Fanny Cazettes[6], Gaelle A Chapuis[2], Anne K Churchland[14], Yang Dan[15], Felicia Davatolhagh[14], Peter Dayan[11], Sophie Denève[16], Eric EJ DeWitt[6], Ling Liang Dong[17], Tatiana Engel[7], Michele Fabbri[1], Mayo Faulkner[8], Robert Fetcho[7], Ila Fiete[17], Charles Findling[2], Laura Freitas-Silva[6], Surya Ganguli[10], Berk Gercek[2], Naureen Ghani[12], Ivan Gordeliy[16], Laura M Haetzel[7], Kenneth D Harris[8], Michael Hausser[18], Naoki Hiratani[4], Sonja Hofer[12], Fei Hu[15], Felix Huber[2], Julia M Huntenburg[11], Cole Hurwitz[1], Anup Khanal[14], Christopher S Krasniak[3], Sanjukta Krishnagopal[4], Michael Krumin[8], Debottam Kundu[11], Agnès Landemard[13], Christopher Langdon[7], Christopher Langfield[1], Inês Laranjeira[6], Peter Latham[4], Petrina Lau[18], Hyun Dong Lee[1], Ari Liu[17], Zachary F Mainen[6], Amalia Makri-Cottington[18], Hernando Martinez-Vergara[12], Brenna McMannon[7], Isaiah McRoberts[5], Guido T Meijer[6], Maxwell Melin[14], Leenoy Meshulam[19], Kim Miller[9], Nathaniel J Miska[12], Catalin Mitelut[1], Zeinab Mohammadi[7], Thomas Mrsic-Flogel[12], Masayoshi Murakami[20], Jean-Paul Noel[5], Kai Nylund[9], Farideh Oloomi[11], Alejandro Pan-Vazquez[7], Liam Paninski[1], Alberto Pezzotta[4], Samuel Picard[8], Jonathan W Pillow[7], Alexandre Pouget[2], Florian Rau[6], Cyrille Rossant[8], Noam Roth[9], Nicholas A Roy[7], Kamron Saniee[1], Rylan Schaeffer[17], Michael M Schartner[6], Yanliang Shi[7], Carolina Soares[12], Karolina Z Socha[13], Cristian Soitu[3], Nicholas A Steinmetz[9], Karel Svoboda[21], Marsa Taheri[14], Charline Tessereau[11], Anne E Urai[3], Erdem Varol[1], Miles J Wells[8], Steven J West[12], Matthew R Whiteway[1], Charles Windolf[1], Olivier Winter[6], Ilana Witten[7], Lauren E Wool[8], Zekai Xu[4], Han Yu[1], Anthony M Zador[3], Yizi Zhang[1]

[1]Zuckerman Institute, Columbia University, New York, NY, USA
[2]Department of Basic Neuroscience, University of Geneva, Geneva, Switzerland
[3]Cold Spring Harbor Laboratory, Cold Spring Harbor, NY, USA
[4]Gatsby Computational Neuroscience Unit, University College London, London, UK
[5]Center for Neural Science, New York University, New York, NY, USA
[6]Champalimaud Center for the Unknown, Lisboa, Portugal
[7]Princeton Neuroscience Institute, Princeton University, Princeton, NJ, USA
[8]Institute of Neurology, University College London, London, UK
[9]Department of Biological Structure, University of Washington, Seattle, WA, USA
[10]Department of Applied Physics, Stanford University, Stanford, CA, USA
[11]Max Planck Institute for Biological Cybernetics, Tübingen, Germany
[12]Sainsbury-Wellcome Centre, University College London, London, UK
[13]Institute of Opthalmology, University College London, London, United Kingdom
[14]Department of Neurobiology, University of California, Los Angeles, CA, USA
[15]Department of Molecular and Cell Biology, University of California, Berkeley, CA, USA
[16]Département D'études Cognitives, École Normale Supérieure, Paris, France
[17]Department of Brain and Cognitive Sciences, Massachusetts Institute of Technology, Cambridge, MA, USA
[18]Wolfson Institute of Biomedical Research, University College London, London, United Kingdom
[19]Center for Computational Neuroscience, University of Washington, Seattle, WA, USA
[20]Department of Physiology, University of Yamanashi, Kofu, Yamanashi, Japan
[21]The Allen Institute for Neural Dynamics, Seattle, Washington, USA
